# Gut Microbial and Associated Metabolite Markers for Colorectal Cancer Diagnosis

**DOI:** 10.3390/microorganisms11082037

**Published:** 2023-08-08

**Authors:** Areej A. Alhhazmi, Renad M. Alhamawi, Reema M. Almisned, Hanouf A. Almutairi, Ahdab A. Jan, Shahad M. Kurdi, Yahya A. Almutawif, Waleed Mohammed-Saeid

**Affiliations:** 1 Medical Laboratories Technology Department, College of Applied Medical Sciences, Taibah University, P.O. Box 344, Al-Madinah Al-Munawarah 42353, Saudi Arabia; rhamawi@taibahu.edu.sa (R.M.A.); msk8lab@gmail.com (S.M.K.); ymutawif@taibahu.edu.sa (Y.A.A.); 2 Seha Polyclinic, P.O. Box 150, Al-Madinah Al-Munawarah 41311, Saudi Arabia; reemaalmisned@gmail.com; 3 Bioscience Program, Biological and Environmental Science and Engineering Division, King Abdullah University of Science and Technology (KAUST), P.O. Box 6900, Thuwal 23955, Saudi Arabia; hanouf.almutairi@kaust.edu.sa; 4 Abdulla Fouad Medical Supplies and Services (AFMS), P.O. Box 150, Al-Madinah Al-Munawarah 21414, Saudi Arabia; ahdab.jan@abdulla-fouad.com; 5 Department of Pharmaceutics and Pharmaceutical Technology, College of Pharmacy, Taibah University, P.O. Box 344, Al-Madinah Al-Munawarah 42353, Saudi Arabia; wneyaz@taibahu.edu.sa

**Keywords:** gut microbiota, colorectal cancer, metabolites, 16s rRNA sequence, real-time PCR, CRC, ADA

## Abstract

Globally, colorectal cancer (CRC) is the second most common cause of mortality worldwide. Considerable evidence indicates that dysbiosis of the gut microbial community and its metabolite secretions play a fundamental role in advanced adenoma (ADA) and CRC development and progression. This study is a systematic review that aims to assess the clinical association between gut microbial markers and/or gut and circulating metabolites with ADA and CRC. Five electronic databases were searched by four independent reviewers. Only controlled trials that compared ADA and/or CRC with healthy control (HC) using either untargeted (16s rRNA gene or whole genome sequencing) or targeted (gene-based real-time PCR) identification methods for gut microbiome profile, or untargeted or targeted metabolite profiling approaches from the gut or serum/plasma, were eligible. Three independent reviewers evaluated the quality of the studies using the *Cochrane Handbook for Systematic Reviews of Interventions*. Twenty-four studies were eligible. We identified strong evidence of two microbial markers *Fusobacterium* and *Porphyromonas* for ADA vs. CRC, and nine microbial markers *Lachnospiraceae*-Lachnoclostridium, *Ruminococcaceae*-Ruminococcus, *Parvimonas* spp., *Parvimonas micra*, Enterobacteriaceae, *Fusobacterium* spp., Bacteroides, *Peptostreptococcus*-*Peptostreptococcus stomatis*, *Clostridia* spp.-*Clostridium hylemonae*, *Clostridium symbiosum*, and *Porphyromonas-Porphyromonas asaccharolytica* for CRC vs. HC. The remaining metabolite marker evidence between the various groups, including ADA vs. HC, ADA vs. HC, and CRC vs. HC, was not of sufficient quality to support additional findings. The identified gut microbial markers can be used in a panel for diagnosing ADA and/or CRC. Further research in the metabolite markers area is needed to evaluate the possibility to use in diagnostic or prognostic markers for colorectal cancer.

## 1. Introduction

Globally, colorectal cancer (CRC) is the most frequently occurring cancer, ranking third in cancer incidence and second in mortality in 2020 and accounting for 1.9 million (10%) new cases and about 935,000 (9.4%) deaths around the world [1]. The rate of CRC incidence varies, with the highest reporting cases in Asia (52.3%) followed by Europe (26.9%) and North America (9.3%). In 2020, there were about 4,007 (14.4%) new cases of CRC in Saudi Arabia, making it the most common cancer [2,3].

CRC is a heterogeneous disease that is usually defined as a carcinoma, mostly an adenocarcinoma (cancer of the glandular tissue) in the colon or rectum. It is formed when healthy cells in the lining of the colon or rectum commence to change and uncontrollably multiply, resulting in the formation of polyps or outgrowths [4]. 

The risk of developing CRC is influenced by many factors, especially environmental and genetic factors. Sex, age, and race are the most crucial elements to be considered in diagnosing CRC. Since colorectal cancer is an illness that is highly affected by gender, males are at a higher risk of developing colorectal cancer, which is approximately 44 percent higher than females [1]. Additionally, between 35 and 40 percent of colorectal cancer cases that are diagnosed have heritable causes, such as low-penetrance genetic mutations, hereditary cancer syndromes like Lynch syndrome, and other unidentified inherited genomic aberrations. With no family history or inherited genomic abnormalities, the remaining 60 to 65 percent of cases are random [1]. 

Microbiota is a complex microbial community that accounts for the integrity of their environment or the well-being of their hosts. The gastrointestinal tract is home to more than 10^14^ microorganisms, which includes almost ten times as many bacterial cells as human cells [5]. Microbiota contributes to many functions in the human body, such as immunological functions, metabolic functions, improving gut integrity, and shaping the intestinal epithelium. In the case of dysbiosis, the changes in microbial composition result in the disruption of these mechanisms [6]. Changes in the microbiota can lead to alteration in human inflammatory status and metabolites-generated by the host and gut-inhabited microbiota, which may directly or indirectly contribute to the etiology of CRC. The gut microbiota is recognized as an essential player in human illnesses such as obesity, inflammatory bowel disease, and colorectal cancer. Advancing facts suggest that microbial dysbiosis is strongly linked with the pathogenesis of intestinal tumors [7]. Recent metagenomics-based research has revealed that *Parvimonas micra*, *Solobacterium moorei*, *Fusobacterium nucleatum*, and *Peptostreptococcus stomatis* have enriched the gut of CRC patients [6]. Furthermore, an increased level of enterotoxigenic *Bacteroides fragilis* has been observed in the colonic mucosa and feces of CRC patients [8,9]. According to the bacterial driver-passenger model for CRC pathogenesis presented by Tjalsma et al. [10], CRC may be started by “driver” bacteria that are then replaced by “passenger” bacteria throughout carcinogenesis. However, it is still unclear how the human gut microbiota contributes to the development of CRC. Understanding the role played by the microbiome in the pathogenesis of CRC is crucial.

An early diagnosis of CRC raises the chances of survival and cure. CRC diagnosis relies largely on colonoscopy, which is an invasive procedure. In addition, performing CRC-specific antigens blood tests to identify carcinoembryonic antigen (CEA) and CA19-9, which are mainly used in the monitoring of CRC patients. One of the highly used tests for the diagnosis of CRC is stool-based tests, for example, gFOBTs which identify the presence of occult blood through the detection of heme pseudo peroxidase activity in the stool. However, the majority of these tests are expensive and exhibit low specificity and sensitivity [11]. Several studies have examined the composition of the gut’s microbes to detect CRC biomarkers and relate certain pathogenic bacteria to CRC, such as *B. fragilis*, *F. nucleatum*, *Streptococcus bovis*, *E. coli*, *Enterococcus faecalis*, and *Porphyromonas* spp. [6]. Given the importance of gut microbiome profiling, which has been extensively conducted using 16S rRNA gene sequencing or shotgun metagenomics techniques [12], the direct link between the gut microbiota at the genus and the species levels, in addition to different CRC stages is challenging. Nevertheless, certain CRC microbial biomarker strains can be easily influenced by diet, antibiotics, hormone treatment, and chemotherapy.

In the case of CRC, disruption to the epithelial and mucous barriers, gastrointestinal inflammation, immunological escape, and genetic/epigenetic changes all work together to directly influence CRC development [8,13]. Numerous disorders, including type 1 diabetes, inflammatory bowel disease (IBD), and breast cancers, have been linked to metabolic changes [14,15,16,17,18]. Additionally, it has been shown that metabolites alter in the colon tissue, urine, serum, and feces of CRC patients as well as in CRC animal models [19,20,21]. Hence, accumulating numbers of metabolic markers have been proposed for CRC diagnosis, encompassing short-chain fatty acids [22], amino acids [23], bile acids (BAs) [24,25], tryptophan (Trp) metabolites [26], and L-carnitine metabolite (trimethylamine N-oxide) [27]. Additionally, few studies have linked gut bacteria dysbiosis to the altered metabolites in CRC. 

This study aims to review relevant publications from five different databases to assemble gut microbial markers, gut metabolites, and circulating metabolites associated with CRC. Then, microbial biomarkers association with metabolites in CRC was collectively assessed. The analyzed data sets included those with stool or tissue microbiome sequencing, metabolomics profiling, and/or association studies examining the association between microbiome dysbiosis and CRC. The microbiome sequencing was either targeted for specific microbes using real-time PCR or untargeted, such as metagenomic sequencing or 16s rRNA gene sequencing. The metabolomics profiling for which targeted and untargeted based analyses using different hyphenated liquid chromatography—mass spectrometric (LC-MS) techniques of gut or plasma/serum samples were included.

## 2. Materials and Methods

**Figure 1:**
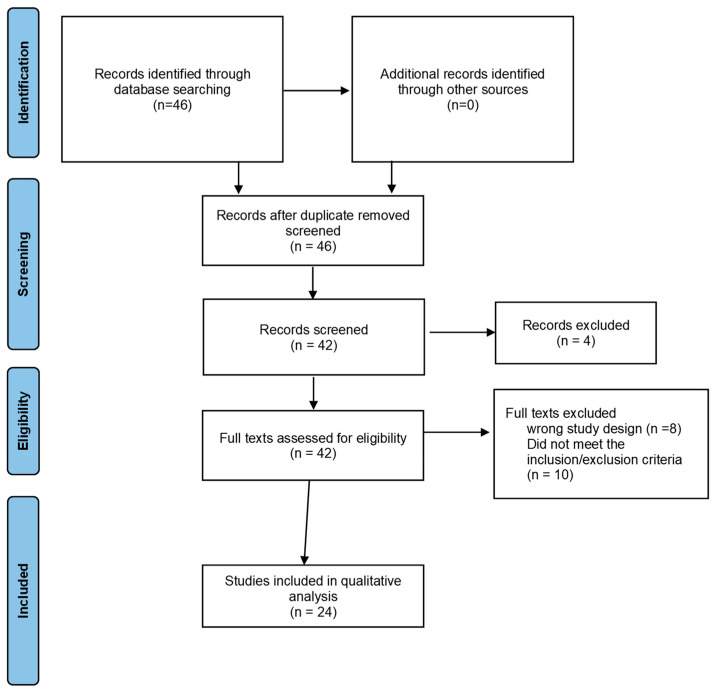
Search strategy guided by the PRISMA flow diagram [29].

In this systematic review of the literature, we used the *Cochrane Handbook for Systematic Reviews of Interventions* and examined the gut microbiota, gut metabolite indicators, and/or circulating metabolite markers as the intervention [28]. Our reporting was planned according to the Preferred Reporting Items for Systematic Reviews and Meta-Analyses (PRISMA) statement [29]. Literature search and study selection: a systematic search was conducted till 30 October 2022, using MEDLINE1, Google Scholar, Wiley, ScienceDirect, and Spring. Three experts (A.A.h, R.M.A, and W.M.S) in the fields of immunology, bioanalytical techniques, and microbiology collaborated to choose the search terms. The references cited in the listed publications were examined to find other studies. Five authors (Y.A.A, R.M.M, AAM, S.M.K, and A.A.J) selected studies that compared healthy controls with adenoma and/or carcinoma with respect to gut microbiome markers and/or gut and/or circulating metabolite markers, and their association for diagnosis or prognosis purposes. Following the selection, three authors (A.A.h, R.M.A, and W.M.S) reviewed the selected papers up until 30 December 2022; results from each database were reviewed, and duplicates were excluded (Figure 1).

The CRC group was defined as cancer patients where cancer starts in the colon or rectum. The development of CRC occurs in stages, starting with normal epithelium, progressing through a pre-malignant lesion (known as an adenoma), into a malignant lesion (carcinoma), which invades nearby tissues and has the potential to spread throughout the body (metastasis). The intervention was identified using the search term “colorectal cancer”, “adenoma”, “carcinoma”, “polyps adenoma”, and “sporadic carcinoma”. The gut or intestinal microbiome was defined as the composition of microorganisms (bacteria, archaea, and eukaryota) colonizing the human gastrointestinal tract. Gut or intestinal microbiome intervention was identified using the search terms “gut or intestinal microbiota”, “gut or intestinal microbiome”, “gut or intestinal microbiome profile”, “gut or intestinal microbiota profile”, “gut or intestinal microbiome markers”, and “gut or intestinal microbiota markers”. Gut or intestinal and circulating metabolites were defined as small molecules that are generated as intermediate or end products of microbial metabolism in the gastrointestinal tract or intestinal and/or circulating system. The intervention was identified using the search term “gut or intestinal metabolites”, “gut or intestinal metabolomic”, “gut or intestinal metabolite profile”, “gut or intestinal metabolomic profile”, “gut or intestinal metabolite markers”, “gut or intestinal metabolomic markers”, “serum metabolites”, “serum metabolomic”, “serum metabolite profile”, “serum metabolomic profile”, “serum metabolite markers”, “serum metabolomic markers”, “plasma metabolites”, “plasma metabolomic”, “plasma metabolite profile”, “plasma metabolomic profile”, “plasma metabolite markers”, “plasma metabolomic markers”.

### 2.1. Eligibility Criteria 

Only studies that compared healthy individuals to people diagnosed with adenoma or carcinoma and underwent peer review were considered. Reports on conference proceedings, case series with less than ten participants, case studies, systematic reviews, and protocol papers were all excluded. Three researchers (AAh, RA, and WMS) with a collective experience of more than ten years in the literature review chose the studies. The complete texts of the potentially suitable studies were retrieved after each title and abstract had been independently reviewed. At the titles and abstracts stage, disagreements were settled by consensus.

### 2.2. Data Extraction 

Based on published guidelines, a standard form (Table S1) was created to retrieve data [30,31,32]. Three researchers (A.A.h, R.M.A, and W.M.S) extracted and cross-checked the data for each study. For each study, the following details were recorded: (1) Participant characteristics, including sample size, age, gender, and diagnosis; (2) Inclusion and Exclusion Criteria; and (3) Interventional features: untargeted; gut microbiome profile, untargeted gut/circulating metabolite profile, the association between gut microbiome species and colorectal cancer, the association between gut/circulating metabolite profile and colorectal cancer, and (4) characteristics of the outcomes: gut microbiome/genera/species, gut/circulating metabolites types.

Based on sensitivity, specificity, and area under the curve (AUC), the diagnostic performance of the investigated biomarkers was evaluated. If any of the data could not be directly described, the appropriate values were, if possible, calculated using other information.

### 2.3. Methodological Quality

The included studies’ quality was evaluated in accordance with PRISMA and the Strengthening the Reporting of Observational Studies in Epidemiology (STROBE) guidelines [30]. The subject recruitment, examiners, methodology, results, handling of missing data, statistical analysis, and findings were the seven categories that were the focus of the quality review (Table S2). Each publication was critically analyzed independently by three reviewers (A.A.h, R.M.H, and W.M.S), and conclusions were confirmed by consensus. Prior to the thorough assessment, five full-text papers were evaluated and discussed for calibration. Studies were given a quality score based on a minimum threshold of 70%; those that met the threshold were deemed to be of good quality, and those that fell below it were assessed to be of low quality [31] (Table 1).

## 3. Results

### 3.1. Studies Included in the Review

After excluding duplicates, the search resulted in 42 references (Figure 1). A title and abstract screening resulted in the exclusion of 18 papers [32,33,34,35,36,37,38,39,40,41,42,43,44,45,46,47,48,49]. As a result, 24 papers in total met the criterion for selection. The most frequent reasons for exclusion were failing to meet the exclusion criteria (e.g., using animals in experiments or simply conducting bioinformatic analyses from databases) or using the incorrect study design (e.g., leaving out the healthy comparison group or CRC).

### 3.2. Comparison Groups/Subgroups of the Studies

Twelve studies included the three basic comparison groups; ADA, CRC, and HC, whereas ten studies included participants from CRC and HC only. Two studies had only two comparison groups, ADA and HC. All studies included both genders except one paper included only male participants, and in four studies, gender was not reported. Age range varied among the included studies, for which the youngest reported age was 18 yrs. Among the included studies, eight papers recorded cancer locations, and nine studies specified cancer stages (Table 2). Table 2 summarizes the study type, recruitment strategy, selection criteria, sample size, study frame time, and location.

### 3.3. Interventions of the Included Studies

Of the 24 studies meeting the inclusion criteria, 11 papers investigated both gut microbiome and associated metabolites, seven papers profiled only gut microbiome, and six described associated metabolites in CRC patients. Thirteen studies conducted an untargeted gut microbiome technique, whereas four performed targeted methods among the included studies. One study performed untargeted microbiome profiling, followed by the targeted method. For metabolites profiling, eight studies employed an untargeted profiling technique, and one study did the untargeted followed by the targeted method. Eight studies used the targeted metabolite method (Table 3).

The majority of the studies (9 out of 11) conducted both microbiome and metabolite profiling using fecal specimens. One study used rectal mucosa biopsy, and another study used stool to extract bacterial extra vesicles (EV). All but one of the seven studies that only focused on microbiome profiling used fecal specimens. The remaining study used rectal mucosa biopsy along with the fecal specimen. For metabolite profiling studies, three studies used fecal specimens, two used serum specimens, and one used plasma specimens. From the resulting 24 studies, we reported the outcome measurement of metabolites as (distribution of metabolite types) (Table 3). Microbiome outcomes were documented as (the distribution of different genera/species in the different study groups and fold change of specific gene expression of particular species). Table 3 summarizes the interventions, the comparison groups, the specimen type, and the metric used in the included studies.

Five studies (Flemer et al. [67], Zeller et al. [68], Zacular et al. [69], Eklöf et al. [71], and Gao et al. [72]) investigated only bacteria as biomarkers and also reported AUCs for diagnostic evaluation. According to Zeller et al. [68], six bacteria differentiated between CRC and healthy controls with an AUC of 85% (84–87%); similarly, Flemer et al. [67] identified six bacteria that distinguished between CRC and healthy controls with an AUC of 87%. Eklöf et al. [71] showed that only one bacterium can differentiate between ADA and CRC with an AUC of 73.1%, yet with 84.6% sensitivity and 63% specificity. Six, four, and six bacteria were used to identify ADA vs. HC, ADA vs. CRC, and CRC vs. HC with AUC values of 79.8% (687–90.8%), 82.3% (72.2–92.3%), and 83.9% (74–93.8%), respectively, as reported by Zacular et al. [69]. Gao et al. [72] showed AUCs of 61.6% (52–71%) (sensitivity: 83.6% and specificity: 39%) and 85.8% (78–93%) (sensitivity: 66.7% and specificity: 98%) for when 18 bacterial species implemented for the diagnosis of ADA or CRC, respectively (Table 4).

Two studies (Yang et al. [75] and Godert et al. [61]) reported only metabolites as bioindicators and evaluated CRC diagnostic implementation. According to Yang et al. [75], two metabolites, cadaverine and putrescine, can be used to identify CRC with AUCs of 77% and 67.2, respectively. An AUC of 77% based on 10 metabolites was reported by Godert et al. [61] (Table 4).

Three studies (Kim et al. [56], Coker et al. [60], and Chen et al. [70]) evaluated the diagnostic application of both biomarkers, bacteria, and metabolites. According to Kim et al. [56], using the identified bacteria alone can have an AUC of 95%, and the two metabolites alone can generate an AUC of 92%; however, combining the two bacteria and the two metabolites improved the AUC to 100%. An AUC of 94.7% (91.5–96.83%) and 87.59% (83.58–91.6%) based on only 6 bacteria and 14 bacteria differentiated between ADA vs. CRC and ADA vs. HC, respectively. However, when the 14 bacteria were combined with the two metabolites, the AUC was 93% (91.07–96.42%) for CRC diagnosis by Coker et al. study [60]. When *Bacteroidetes* was combined with Acetic acid, butyric acid, and *t10*, *c12-CLA*, they exhibited an AUC of 90% (70–90%) to differentiate prelesion (ADA) as Chen et al. [70] reported (Table 4).

### 3.4. Methodological Quality

Sixteen studies met the methodological high-quality threshold of 70% (Table 5) [26,50,52,54,56,57,58,60,62,63,66,67,68,69,70,75]. Four studies scored between 60 and 69% [71,72,74,75], and four studies scored 50–59% [53,59,61,73]. The major source of bias in the resulting 24 papers was the failure to report whether the person(s) experimenting was/were blinded to the study groups and quality controls, followed by the statistical analyses used, such as reporting the confidence interval for change in outcomes from before to after intervention, the distribution of principal confounders in each group of subjects, and adjustment for confounders in the analyses. All studies noticeably described (1) their sample size estimation for each experimental group, (2) their main findings, and (3) the main hypothesis and objectives and validity of the reported main outcome.

### 3.5. Measurement Outcomes

#### 3.5.1. Primary Outcome Measures

##### Microbial Markers among ADA and CRC Compared to Healthy Control (HC) Using the Untargeted Microbiome Approach

Microbial markers associated with CRC and ADA were evaluated in 18 studies by two approaches: untargeted or targeted method. The untargeted approach applied either 16s rRNA gene or whole genome sequencing analysis, whereas the targeted method used real-time PCR targeting specific microbial genes. Eleven studies used the 16s rRNA gene sequencing analysis [26,50,56,62,63,67,69,70,72,74,75], and two studies used the whole genome sequencing analysis [53,60,68] (Table 3).

There was conflicting evidence of microbial markers between ADA and HC (Nugent et al. [52], Zackular et al. [69], Chen et al. [70], Gao et al. [72]). However, there was strong evidence of associated microbial markers for CRC compared to ADA. Two microbial markers were found to be increased in CRC compared to ADA, *Fusobacterium* spp. (Zeller, et al. [68], Zackular et al. [69], and Gao et al. [72]) and *Porphyromonas* (Zeller et al. [68] and Zackular et al. [69]. *Fusobacterium* spp. was identified in two high-quality studies (Zeller et al. [68] and Zackular et al. [69]) and one moderate-quality paper (Gao et al. [72]). *Porphyromonas* was profiled in two high-quality papers (Zeller et al. [68], Zackular et al. [69]) (Table 6a).

There was strong evidence that nine microbial markers were associated with CRC compared to HC as follows: *Lachnospiraceae-Lachnoclostridium*, Ruminococcaceae-*Ruminococcus*, *Parvimonas* spp., *P micra*, Enterobacteriaceae, *Fusobacterium* spp., *Bacteroides*, *Peptstreptococcus*-*P. stomatis*, *Clostridia* spp.-*Clostridium hylemonae*, *Clostridium symbiosum*, and *Porphyromonas-P. asaccharolytica* (Table 6a).

Lachnospiraceae-*Lachnoclostridium* and Ruminococcaceae-*Ruminococcus* were identified in three high-quality papers: Kim et al. [56], Sinha et al. [62], and Zackular et al. [69] and Kim et al. [56], Flemer et al. [67] and Zeller, et al. [68], respectively. *Parvimonas* spp.-*P. micra* was profiled in three high-quality studies (Kim et al. [56], Flemer et al. [67], and Zeller et al. [68]) and one in a moderate-quality study (Gao et al. [72]). The group Enterobacteriaceae was found as microbial markers in CRC patients in three high-quality studies (Kim et al. [56], Zackular et al. [69], and Yang et al. [75]) (Table 6a).

*Fusobacterium* is one of the most common CRC-microbial markers, five high-quality papers (Shina et al. [62] Flemer et al. [67], Zackuler et al. [69] and Yang et al. [75]) and one moderate-quality study (Gao et al. [72]) identified this genus. Zeller et al. [68] typed *Fusombacterium* to the sub-species as *F. nucleatum* subsp. *vincentii*, *F. nucleatum* subsp. *Animalis*, *Fu. nucleatum* subsp. *nucleatum*, *F. nucleatum* subsp. *Polymorphum*, whereas Gao et al. [72] identified the species level only *F. nucleatum* (Table 6a).

*Bacteroids* were profiled in two high-quality papers (Zeller et al. [68] and Felmer et al. [67]), whereas in Zeller et al. [68] specifically *B. fragilis* was characterized. *P. stomatis* is another CRC-microbial marker that was described in two high-quality studies (Felmer et al. [67] and Zeller et al. [68]) and one low-quality paper (Gao et al. [72]). *Clostridia* spp. was characterized in two high-quality papers (Shinan et al. [62] and Zeller et al. [68]), where two species, *C. hylemonae*, *C. symbiosum*, were described in Zeller et al. [68]. *Porphyromonas* was profiled as a CRC-microbial marker in two high-quality studies (Zeller et al. [68] and Zackular), in Zeller et al. [68] *P. asaccharolytica* was identified (Table 6a).

There was limited evidence of the association of Streptococcus spp. with CRC compared to HC, as the two studies profiled *Streptococcus* spp. were in the low-quality category. Chang et al. [53] identified *S. gallolyticus* and another study (Goa et al. [72]) described S. intermedius. Results indicated no evidence of the association of the other microbial markers shown in Table 6a with CRC compared to HC.

##### Microbial Markers among ADA and CRC Compared to Healthy Control (HC) Using the Targeted Microbiome Approach

Microbial markers associated with CRC and ADA were evaluated in four studies using real-time PCR targeting specific microbial genes. No studies identified microbial markers associated with ADA compared to HC and ADA compared to CRC. However, there was moderate evidence of *Fusobacterium* spp.-*F. nucleatum* as a microbial marker for CRC compared to HC. Two studies characterized *Fusobacterium* spp. as a microbial marker, one with high-quality (Clos-Garcia et al. [63]) and one with a low-quality score (Eklöf et al. [71]) (Table 6b).

##### Metabolite Markers among ADA and CRC Compared to Healthy Control (HC) Using the Non-Targeted and Targeted Metabolite Approaches

Metabolite markers linked with CRC and ADA were assessed in 17 studies in two ways, non-targeted or targeted profiling methods. The non-targeted approach applied (1 study [50]) Ultra-Performance Liquid Chromatography/Mass Spectrometry platform (UPLC-MS/MS), (1 study [52]) Liquid chromatography coupled to Gas Chromatography Time-of-Flight Mass Spectrometry (LC-GCTOF-MS/MS), (1 study [56]) Gas Chromatography Time-of-Flight Mass Spectrometry (GCTOF-MS/MS), (1 studies [61]) High-Performance Liquid Chromatography/Mass Spectrometry platform (HLC-MS/MS), (2 studies [74,75]) Gas Chromatography—Mass Spectrometry (GC-MS), (1 study [62]) HPLC-GC-MS/MS analyses, (1 study [66]) GCTOF-MS-UPLC-QTOF-MS, and (1 study [70]) Ion Chromatography/UPLC-MS/MS. The targeted approach varied among the nine studies: (2 studies [26,63]) UPLC-MS/MS, (1 study [54]) LC-MS/MS, (2 studies [57,74]) GC-MS/MS, (2 studies [58,73]) GC, (1 study [60]) GCTOF-MS/MS, and (1 study [22]) HPLC platforms (Table 3).

There was conflicting evidence of common metabolite markers in ADA compared to HC. Three studies (Kim et al. (high-quality) [56], Nugent et al. (low-quality) [52], and Kim et al. (high-quality) [50]) identified metabolite markers in ADA compared to the HC group using the untargeted means.

There was limited evidence of one metabolite marker (Palmitoyl–sphingomyelin) linked to CRC compared to HC [61,62], whereas there was moderate evidence of another metabolite marker, Proline [66,74], associated with CRC compared to HC. Palmitoyl-sphingomyelin was profiled in two papers, a high-quality paper [62] and a low-quality study [61]. The amino acid, Proline, was identified in a high-quality study [66] and low-quality paper [74] (Table 6c).

Only one study identified metabolite markers using the targeted method for ADA vs. HC groups or ADA vs. CRC groups. Seven studies profiled metabolite markers in CRC vs. HC [26,54,57,58,60,73,75], yet there were conflicting results (no common markers). Three high-quality papers [26,54,60] and four studies of low-quality [57,58,73,75] identified the metabolite markers (Table 6d).

#### 3.5.2. Secondary Outcome Measures

##### Microbial Markers for Cancer Stages and Locations

Among the included studies, eight papers recorded cancer locations, and nine studies specified cancer stages (Table 3). Based on the untargeted means, one paper [72] identified microbial markers for early stage I, III, and late stage IV. Moreover, one paper [67] profiled microbial markers for different cancer locations. There was no evidence of distinguished microbial markers among the different stages or locations. On the targeted approach, one paper [63] described microbial markers for late-stage IV. Moreover, one paper [22] profiled microbial markers for cancer on the left side. There was no evidence of distinguished microbial markers among the different stages or locations.

## 4. Discussion

The present systematic review identified strong evidence of two microbial markers for CRC compared to ADA; *Fusobacterium* spp.-*F. nucletaum* (Zelleret al. [68], Zackular et al. [69], and Gao et al. [72]) and *Porphyromonas* (Zeller et al. [68] and Zackular et al. [69]) using the untargeted interventions. Yet, using the targeted method, no evidence was identified for microbial markers associated with CRC compared to ADA.

We identified strong evidence of nine microbial markers associated with CRC compared to HC as follows: Lachnospiraceae-Lachnoclostridium, Ruminococcaceae-*Ruminococcus*, *Parvimonas* spp., *P. micra*, Enterobacteriaceae, *Fusobacterium* spp., *Bacteroides*, *Peptostreptococcus*-*P. stomatis*, *Clostridia* spp.-*C. hylemonae*, *C. symbiosum*, and *Porphyromonas-P. asaccharolytica* using the untargeted approach. Moreover, results indicated moderate evidence of *Fusobacterium* spp.-*F. nucleatum* as a microbial marker for CRC compared to HC. However, we could not identify evidence for any microbial markers associated with ADA compared to HC using the untargeted and targeted methods.

These findings are consistent with the findings of a systematic review conducted by Russ et al., which investigated the association between the human gut microbiome and the risk of CRC. The study found that *Fusobacterium* and *Bacteroides* were the most enriched microbial species in CRC compared to HC [76]. Another systematic review found nine fecal microbiotas (*Fusobacterium*, *Enterococcus*, *Porphyromonas*, *Salmonella*, *Pseudomonas*, *Peptostreptococcus*, *Actinomyces*, *Bifidobacterium,* and *Roseburia*) to be associated with colorectal neoplasia [77].

In the current systematic review, results indicated conflicting evidence of metabolite markers for ADA in comparison to HC using the untargeted methods, yet no evidence using the targeted approach. Limited evidence was demonstrated of Palmitoyl–sphingomyelin as a metabolite marker of CRC compared to HC [61,62], whereas moderate evidence was identified of an amino acid, Proline [66,74], as a metabolite marker for CRC compared to HC using the untargeted approach. However, results demonstrated conflicting evidence of associated metabolite markers with CRC vs. HC using the targeted intervention. There was no evidence of distinguished metabolite markers for ADA compared to CRC using both untargeted and targeted interventions.

The enrichment of amino acids, cadaverine, and creatine in CRC was discovered by a recent meta-analysis that combined LEfSe, random forest (RF), and cooccurrence network approaches to find a collection of global CRC biomarkers. They had a positive correlation with microorganisms linked to CRC (*P. stomatis*, *Gemella morbillorum*, *B. fragilis*, Parvimonas species, *F. nucleatum*, *Solobacterium moorei*, and *Clostridium symbiosum*), but their correlation with microbes linked to controls was negative [6].

Secondary outcomes were not frequently used in the included studies, with no microbial or metabolite fingerprint for the different groups. These included microbial and metabolite markers for cancer stages and cancer locations. Based on the evidence investigated here, no evidence was identified of microbial or metabolite markers for the ADA vs. HC, ADA vs. CRC, or CRC vs. HC using targeted or untargeted interventions. Based on these studies, further investigation of the outcomes in relation to the ADA and CRC is warranted.

## 5. Study Limitations

Studies only available in English were included in this review; no search of the grey literature was performed. A potential bias in the choice of pertinent studies may have resulted from three sources. As the publications included in this systematic review varied greatly in their methodological approaches, comparison groups, and statistical analyses, meta-analysis was not possible. Gut microbiome and associated metabolites are subjected to confounding variables such as age, gender, diet, medication, smoking, and other lifestyle factors [78]. Moreover, there can be significant differences in the gut microbiome and its metabolites between geographically distinct populations and across countries [79,80].

More than 83% of the included studies focused primarily on identifying biomarkers for CRC diagnosis, yet four studies (16.6%), particularly Sun et al. [26], Nugent et al. [52], Flemer et al. [67], and Yusuf et al. [73], the main aim was to identify microbes or metabolites that could contribute to the pathology of CRC. Sun et al. [26] study identified bacteria and metabolites; Nugent et al. [33] reported associated bacteria with CRC; Flemer et al. [67]; and Yusuf et al. [73] studied only associated metabolites. These papers included healthy controls in comparison to ADA or CRC and performed association analysis to evaluate the contribution of such markers in the CRC progression, suggesting these microbes or metabolites as potential markers of CRC diagnosis. Therefore, we included the four studies in the analysis. However, further evaluation from a diagnostic perspective is much needed.

Various alpha and beta indices, including the Bray–Curtis dissimilarity, Jaccard distance, and UniFrac, as well as the Chao Index, Simpson Index, Shannon Index, ACE Index, and Good’s Coverage Index, have been reported across the included research. Most of the studies that were considered demonstrated microbial dysbiosis between CRC and the healthy control group. The stated estimates for alpha and beta diversity are indices rather than true effective difference figures. Due to the non-linear nature of these indices, it is incorrect to compare them between different studies and draw inferences about their biological importance. Therefore, we have not reported and compared these indices in the systematic review.

Most of the included studies were conducted in Asian countries (Table 2), which can be untransferable across the world. Additionally, depending on the interventions used in this research, some of our specific summary statements were in disagreement with one another. (Table 6). There was no consistency in sample types, collection, and storage temperature. Moreover, the lack of standardization in DNA and metabolite extractions across the included studies has influenced microbiome and metabolite profiling. Further, one of the major conflicts observed was for the intervention approaches, untargeted and targeted methods. Each method applied different analytical means. Microbiome profiling used either 16S rRNA gene or whole genome sequencing for an untargeted approach, or real-time PCR for a targeted approach. Each method has its limitations from the taxonomic analysis perspective [81]. Likewise, metabolite profiling was conducted by a variety of methods. There was significant variation among these methodologies, which could lead to biases and make comparisons between the groups difficult. [82]. Therefore, the level of evidence assessment was classified into two main categories: the untargeted and targeted approaches for each microbial and metabolite profile. There were three studies with low quality (weighted 51.8%, 55.5%, and 59.3% in the summary statement, respectively). This suggests that even a different observation from a low-quality study could substantially alter the strength of the evidence for a given summary conclusion. This might have made it more difficult to distinguish between fingerprint marks left by different groups and caused frequent inconsistencies in evidence summary statements.

## 6. Conclusions

We identified strong evidence of two microbial markers, *Fusobacterium* spp.-*F. nucletaum* and *Porphyromonas* for ADA vs. CRC, and nine microbial markers *Lachnospiraceae-Lachnoclostridium*, *Ruminococcaceae-Ruminococcus*, *Parvimonas* spp., *P. micra*, Enterobacteriaceae, *Fusobacterium* spp., *Bacteroides*, *Peptostreptococcus*-*P. stomatis*, *Clostridia* spp.-*C. hylemonae*, *Clostridium symbiosum*, and *Porphyromonas-P. asaccharolytica* for CRC vs. HC.

Based on the data that have already been reviewed here, there is encouraging evidence that microbial markers from fecal samples may be used to develop new, inexpensive tests that could supplement the collection of existing non-invasive CRC screening tools. However, to make results more comparable and allow for the drawing of conclusions on a wider scale, future research should concentrate on creating standardized and reproducible protocols for researching the human gut microbiota.

The remaining evidence of metabolite markers among the different groups ADA vs. HC, ADA vs. HC, and CRC vs. HC was not of sufficiently high quality to permit further conclusions. With this finding, these microbial markers can be used in a panel for the diagnosis of ADA and CRC. Further research in the metabolite markers area is needed to evaluate the possibility of diagnostic or prognostic markers for colorectal cancer.

## Figures and Tables

**Table 1:** Levels of evidence for summary statements and description of criteria adopted a priori to determine the level of evidence.

Level	Description
Strong	Consistent results (≥70%) from at least 2 high-quality studies
Moderate	1 high-quality study and consistent findings (≥70%) in 1 or more low-quality studies
Limited	Findings in 1 high-quality * study or consistent results (≥70%) among low-quality studies
NO	No study identified
Conflicting	Inconsistent results, irrespective of study quality

* Studies with quality scores over 70% were deemed high quality.

**Table 2:** Description of study type and study participants in the included studies.

Author	Study Type	Recruitment Strategy and Selection Criteria	Number of Subjects and Groups				Location and Time Frame
Sun et al. [26]	Case-control study for untargeted microbiome and targeted metabolites identification, specifically Tryptophan and its metabolites in CRC patients	Male and female Aged 18–80 yrs ADA, CRC, HC	Healthy control = 38 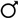 24 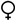 14 56.85 yrs ± 10.99	ADA = 33 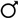 23 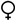 10 61.18 yrs ± 8.53		CRC = 46 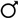 32 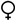 14 63.63 yrs ± 11.39	The China–Japan Friendship Hospital, China March 2019 and December 2019
Kim et al. [50]	Case-control study for untargeted metabolites and microbiome identification in CRC patients Ps. The samples were obtained from cross sectional study, which gives this study a cross-sectional nature	All samples selected here have been enrolled in previous study [51] Male and female Aged 50–80 yrs ADA, CRC, and HC.	Healthy control = 102 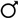 62 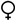 40 50–59 yrs = 18 60–69 yrs = 49 >70 yrs = 35	ADA = 102 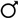 62 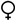 40 50–59 yrs = 17 60–69 yrs = 50 >70 yrs = 35		CRC = 6 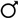 20 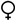 16 50–59 yrs = 6 60–69 yrs = 19 >70 yrs = 11	ND 2001 to 2007
Nugent et al. [52]	Case-control study for targeted microbiota (*Lactobacillus* sp., *Escherichia coli*, *Bifidobacterium* sp., *Clostridium* sp., *Bacteroides* sp., and *Eubacteria*) and untargeted metabolites identification in CRC patients	Male and female Aged > 30 yrs ADA and HC	Healthy control = 15 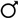 4 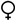 11 55.0 yrs ± 1.1		ADA = 15 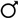 6 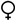 9 54.3 yrs ± 1.1		University of North Carolina Hospitals, USA ND
Chang et al. [53]	Case-control study for untargeted microbiome in CRC patients	Only Male Aged 38–77 yrs CRC and HC	Healthy control = 12 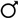 12		CRC = 6 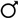 6		Haikou people’s Hospital, Hainan, China ND
			Metagenomics sequences of 59 patients with CRC were obtained from the NCBI database (ref_CRC, Metagenomics sequencing data: PRJEB7774).				
Guertin et al. [54]	Case-control study for targeted metabolites, trimethylamine N-oxide, Carnitine, Choline, and Betaine in CRC patients “Nested case-control study within the Alpha Tocopherol and Beta Carotene Cancer Prevention (ATBC) Study, described in detail elsewhere [55]	Gender ND Aged 50–69 yrs CRC and HC	Healthy control = 644		CRC = 644		USA ATBC study (1985–1988)–(1993) [55]
			**Tumor location** Proximal colon = 169 Distal colon = 153 Rectum ICD-9 = 282				
Kim et al. [56]	Case-control study for untargeted microbiome and untargeted metabolites in CRC patients	Male and female Aged 45–80 yrs CRC and HC	Healthy control = 40 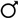 22 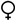 18 49–78 yrs		CRC = 32 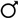 20 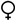 16 45–80 yrs		CRC patients from Seoul National University Bundang Hospital and Chung-Ang University Hospital, South Korea HC individuals from Haewoondae Baek Hospital, South Korea April 2016–April 2018.
			**Tumor Stage** 0 = 1 I = 7 II = 12 III = 9 IV = 3				
			**Tumor location** Cesum = 2 Ascending = 6 Transverse = 1 Sigmoid = 12 Rectal = 7				
Song et al. [57]	Pilot, case-control study for targeted metabolites, long and short fatty acid in CRC patients	Male and female Aged 45–70 yrs ADA, CRC, and HC	Healthy control = 28 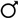 22 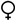 6 51.1 yrs ± 6.0	ADA = 27 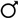 25 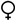 1 53.6 yrs ± 7.2		CRC = 26 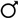 16 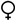 10 59.7 yrs ± 12.2	Asan Institute for Life Sciences, University of Ulsan College of Medicine, South Korea July 2014 and August 2014
			**Tumor stage** I = 3 IIa = 5 IIc = 1 IIIb = 11 IIIc = 3 IVa = 3				
			Presence of lymph node metastasis = 16 Presence of colonoscopic obstruction = 5 **Tumor location** Proximal cancer (above splenic flexure) = 3 Distal cancer (below splenic flexure) = 23				
Genua et al. [58]	Case-control study for targeted metabolites, Acetic Acid, Propionic Acid, i-Butyric Acid, Butyric Acid, 2-MethylButyric Acid, i-Valeric Acid, Valeric Acid from serum in CRC patients	Male and female Cohort Irish and Czech Aged 45–70 yrs Tubular tubulovillous adenoma (TA/TVA), High-grade dysplasia (HGD), CRC, and HC	**Irish cohort 128**				The Adelaide & Meath Hospital in Dublin, Ireland Thomayer Hospital in Prague, Czech Republic.
			Healthy control = 36 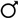 17 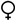 19 58 yrs ± 7	TA/TVA = 48 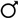 30 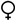 18 61.5 yrs ± 11	HGD = 18 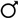 11 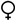 7 59 yrs ± 7	CRC = 26 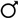 13 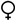 13 56 yrs ± 23	
			**Czech cohort 85**				
			Healthy control = 27 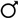 12 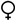 15 56 yrs ± 10		CRC = 58 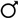 40 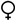 18 64 yrs ± 15		
D’asheesh et al. [59]	Case-control study for targeted microbiota *Lactobaccilus acidophilus*, *Lactobacillus Plantarum*, and *Enterococcus faecalis*	Aged 20–76 yrs Gender ND CRC and HC	Healthy control = 300 45.3 ± 2.5		CRC = 30055.34 ± 3.66		Iran March 2014 to October 2019
Coker et al. [60]	Case-control study for untargeted microbiome and targeted metabolites	Male and female Aged 58–83 yrs ADA, CRC, and HC	Healthy control 128 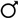 59 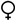 69 64.03 yrs ± 6.84	ADA 140 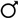 64 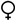 54 65.84 yrs ± 5.53		CRC 118 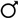 64 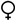 54 73.21 yrs ± 10.37	Prince of Wales Hospital, the Chinese University of Hong Kong ND
Goedert et al. [61]	Case-control study for untargeted metabolites	Male and female Aged 46–75 yrs CRC and HC	Healthy control 102 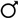 55.9% 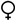 44.1% 58.3 yrs ± 12.9		CRC 48 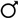 64.6% 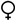 35.4% 62.9 yrs ± 13.7		1985–1989 Washington DC area hospitals, USA
			**Tumor stage** Non-invasive = 20.8% Invasive, no known metastases = 41.7% Known metastases = 35.4% Missing = 2.1%				
			**Tumor location** Right colon = 29.1% Left colon = 33.3% Rectal = 27.1% Missing = 10.4%				
Sinha et al. [62]	Case-control study for untargeted microbiome and untargetd metabolites	Male and female Aged 45–76 yrs CRC and HC	Healthy control = 89 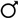 55.5% 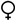 40.5% 58.4 yrs ± 13		CRC = 42 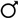 59.5% 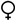 40.5% 63.4 yrs ± 13.1		ND 1985–1987
			**Tumor stage** Non-invasive = 21.4% Invasive, no known metastases = 42.9% Known metastases = 33.3% Missing = 2.1%				
Clos-Garcia et al. [63]	Case-control study for targeted metabolites as in [64] and untargeted microbiome identification in CRC patients	Male and female Aged >18 yrs ADA, CRC, and HC	Healthy control = 77 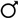 35 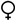 48 64.62 yrs	ADA = 69 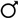 41 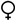 41 67.99 yrs		CRC = 99 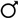 60 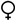 39 70.16 yrs	Samples batch 1 and 2 from COLONPREDICT study [65] Batch 3 from Instituto de Investigación Sanitario Galicia Sur, Spain ND
Tan et al. [66]	Case-control study for untargeted metabolites in CRC patients	CRC and HC Aged 24–82 yrs	Healthy control = 102 31–76 yrs		CRC = 101 24–82 yrs		The Ruijin Hospital affiliated with Shanghai Jiao Tong University School of Medicine, China ND
			**Tumor stage** I = 26 II = 43 III = 26 IV = 6				
			**Tumor location** Ascending = 21 Descending = 9 Sigmoid colon = 7 Rectum = 63				
Flemer et al. [67]	Case-control study for untargeted microbiome from stool and mucosa in CRC patients	Female and male Aged 27–82 yrs CRC, ADA, and HC	Healthy control = 56	Polyps ADA = 21		CRC = 59	Mercy University Hospital, Ireland ND
Zeller et al. [68]	Case-control study for untargeted microbiome from stool and mucosa in CRC patients	Female and male Aged 34–69 yrs Adenoma (small < 1 cm and large > 1 cm) HC from different cohorts from France and Germany	Healthy control = 358 Cohort France = 61 Cohort Germany = 297	ADA = 42 Cohort France ADA small = 27 ADA large = 15		CRC = 91	F group Assistance Publique-Hôpitaux de Paris (academic hospitals) G population the Department of Surgery at the University Hospital Heidelberg and the affiliated Hospital Salem H population From my microbe project http://my.microbes.eu/ (accessed on 12 June 2023) ND
			**Cohort France = 61** **Tumor stage** 0 = 0 I = 15 II = 7 III = 10 IV = 21		**Cohort Germany = 38** **Tumor stage** 0 = 25 I = 0 II = 0 III = 13 IV = 0		
Zackular et al. [69]	Case-control study for untargeted microbiome from stool in CRC patients	Male and female Aged >18 yrs ADA, CRC, and HC	Healthy control = 30 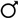 11 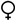 19 55.3 yrs (±9.2)	ADA = 30 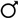 18 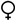 12 61.3 yrs (±11.1)		CRC = 30 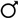 21 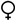 9 59.4 yrs (±11)	Toronto (Canada), Boston (USA), Houston (USA), and Ann Arbor (USA) ND
Ohigashi et al. [22]	Case-control study for targeted metabolites and microbiome from stool in CRC patients	Male and female Aged 52–81 yrs ADA, CRC, and HC	Healthy control = 27 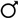 16 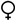 11 65.6 yrs ± 13.5	ADA = 22 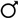 11 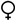 11 66.6 yrs ± 9.2		CRC = 93 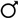 49 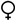 44 68.9 yrs ± 12.1	ND November 2007–October 2010
			**Tumor stage** Dukes A (36 patients) Dukes B (19 patients) Dukes C (24 patients) Dukes D (14 patients)				
Chen et al. [70]	Case-control study for untargeted metabolites and microbiome, followed by targeted microbiota using functional genes from stool in CRC patients	Male and female Aged 40–63 yrs ADA and HC	Healthy control = 30 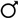 13 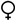 17 50.33 yrs ± 10.87		ADA = 30 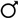 20 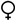 10 53.23 yrs ± 10.14		The First Affiliated Hospital of Kunming Medical University, China November 2017 to April 2018
Eklöf et al. [71]	Case-control study for targeted microbiome in CRC patients	Male and female Aged > 34 yrs CRC, ADA, HC	Healthy control = 65 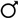 35 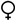 30 34–80 yrs	Dysplasia ADA = 134 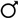 80 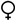 54 34–80 yrs		CRC = 39 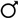 20 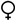 19 34–80 yrs	The University Hospital in Umeå, Sweden September 2008 to March 2013
			**Tumor stage** I = 2 II = 21 III = 8 IV = 7				
			**Tumor location**				
				Total	Dysplasia	CRC	
			Right	37	12	49	
			Left	59	17	76	
			Rectum	38	10	40	
Gao et al. [72]	Case-control study for untargeted microbiome in CRC patients	Male and female Aged ND CRC, precancer (ADA), HC	Healthy control = 442 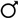 60.65% 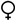 39.35% 65.79 yrs ± 12.73	Precancer (ADA) = 195 (31) 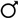 62.5% 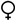 37. 5% 63.07 yrs ± 12.84		CRC = 155 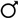 29.48% 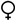 70.52% 64.96 yrs ± 10.44	The Shanghai Tenth People’s Hospital, Tongji University School of Medicine and Changzheng Hospital affiliated with the Naval Medical University, China The discovery cohort from January 2014–November 2015 The validation cohort from March 2016–December 2017
			**Tumor stage** 0 = 25 (16.13%) I = 51 (32.9%) II = 56 (36.13%) III = 11.7 (10%) IV = 12 (7.74%)				
			**Tumor location** Ascending colon = 25 (16.13%) Transverse colon = 7 (4.52%) Descending colon = 10 (6.45%) Sigmoid colon = 33 (21.29%) Rectum = 70 (45.16%) Undefined = 5 (2.3%)				
Yusuf et al. [73]	Case-control study for targeted metabolites, short-chain fatty acids, acetate, propionate and butyrate acids in CRC patients	Male and female Aged >18 yrs CRC and HC	Healthy control = 14 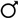 9 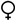 5 50 yrs ± 17.6		CRC = 14 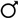 10 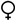 4 53.8 yrs ± 13.3		General Teaching Hospital Banda Aceh, Indonesia ND
Weir et al. [74]	Case-control study for untargeted microbiome and untargeted metabolites followed by targeted for short chain fatty acids in CRC patients	Male and female Aged >18 yrs CRC and HC	Healthy control = 11 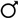 7 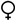 3 50 yrs ± 17.6		CRC = 10 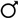 8 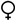 2 53.8 yrs ± 13.3		The University of Colorado Health-Poudre Valley Hospital in Fort Collins, CO, USA ND
			Tumor stage * T1 = 2 T2 = 3 T3 = 4 Tis = 1 * Tis: Carcinoma in situ: intraepithelial or invasion of lamina propria; T1: Tumor invades submucosa; T2: Tumor invades muscularis propria; T3:Tumor invades through muscularis propria into the subserosa or into nonperitonealized pericolic or perirectal tissue.				
			**Tumor location** Ascending 3 Rectum 3 Sigmoid 4				
Yang et al. [75]	Case-control study for untargeted microbiome and metabolites in CRC patients	Male and female Aged >60 and <60 yrs CRC and HC	Healthy control = 50 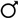 17 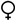 33 >60 yrs = 33 <60 yrs = 17		CRC = 50 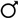 26 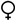 24 >60 yrs = 24 <60 yrs = 26		Ongji University Affiliated Tenth People’s Hospital (Shanghai, China) January 2014 to September 2014

**Table 3:** Description of the intervention used in the included studies.

Author	Group	Intervention		Sample Type	Metric
Sun et al. [26]	Experimental group AD and CRC Control group	Targeted metabolites identification	Untargeted microbiome identification	Fecal specimen	+/− of Trp and its metabolites Indole/Trap ratio Distribution (abundance) at bacterial genera level
		Tryptophan (Trap) and its metabolites, such as L-Trp, L-Kynurenine (KYN), indole, skatole, indole-3-carboxylic acid (I3CA), Indole-3-aldehyde (IALD), Indole-3-acetate (IAA), Indolepropionic acid (IPA), indoxyl-3-sulfate (I3S), and Indole-3-acetadehyde (IAALD) using Ultraperformance liquid chromatography coupled to tandem mass spectrometry (UPLC-MS/MS) analysis	16S geneRNA gene sequencing using an Illumina NovaSeq PE250		
Kim et al. [50]	Experimental group AD and CRC Control group	Untargeted metabolites identification	Untargeted microbiome identification	Fecal specimen	Distribution (abundances) of metabolites Distribution (abundance) bacterial genera
		UPLC-MS/MS platform	16S gene RNA gene sequencing using the Illumina MiSeq system		
Nugent et al. [52]	Experimental group AD Control group	Untargeted metabolites identification	Targeted microbiome identification	Rectal mucosal biopsy	+/− of metabolites Distribution (abundance) of bacterial genera/species
		Liquid chromatography and gas chromatography time of flight mass spectrometry	For *Lactobacillus* sp., *Escherichia coli*, *Bifidobacterium* sp., *Clostridium* sp., *Bacteroides* sp., and *Eubacteria* using qPCR with primers that amplify 16S rDNA		
Chang et al. [53]	Experimental group CRC Control group	Untargeted microbiome identification		Fecal specimen	Distribution (abundance) of bacterial species
		Whole-genome shotgun sequencing Illumina HiSeq			
Guertin et al. [54]	Experimental group CRC Control group	Targeted metabolites identification		Serum specimen	+/− of serum metabolites, trimethylamine N-oxide, Carnitine, Choline, and Betaine Odds ratio of serum metabolites, trimethylamine N-oxide, Carnitine, Choline, and Betaine
		Trimethylamine N-oxide, Carnitine, Choline, and Betaine in CRC patients using liquid chromatography (LC) tend mass spectrometry (MS)			
Kim et al. [56]	Experimental group CRC Control group	Untargeted metabolites identification	Untargeted microbiome identification	Stool to extract bacterial extra vesicles (EV)	Distribution (Abundance) of metabolites Fold change difference of the means Distribution of bacterial genera
		Gas chromatography-time-of-flight mass spectrometry	16S gene RNA gene sequencing by MiSeq Illumina.		
Song et al. [57]	Experimental group CRC Control group	Targeted metabolites identification		Fecal specimen	Distribution (Abundance) of metabolites Mean ± SD
		Long and short fatty acids using gas chromatography—mass spectrometry			
Genua et al. [58]	Experimental group TA/TVA HGD CRC Control group	Targeted metabolites identification		Plasma specimen	+/− of the following metabolites, Acetic Acid, Propionic Acid, i-Butyric Acid, Butyric Acid, 2-MethylButyric Acid, i-Valeric Acid, Valeric Acid Mean/IQ
		Acetic Acid, Propionic Acid, i-Butyric Acid, Butyric Acid, 2-MethylButyric Acid, i-Valeric Acid, Valeric Acid using gas chromatography			
D’asheesh et al. [59]	Experimental group CRC Control group	Targeted microbiome identification		Fecal specimen	Fold change and CFU/ml
		*Lactobacillus acidophilus*, *Lactobacillus palntarom* and *Enterococcus faecalis* By real-time PCR			
Coker et al. [60]	Experimental group ADA and CRC Control group	Targeted metabolites identification	Untargeted microbiome identification	Fecal specimen	Distribution (Abundance) of metabolites Fold change Distribution (Abundance) of bacterial species
		Methyl and ethyl chloroformate (MCF and ECF) derivatized compounds identified previously using gas chromatography coupled to time-of-flight mass spectrometer (GC-TOFMS) analysis	Whole-genome shotgun sequencing of all samples was carried out on an Illumina HiSeq.		
Goedert et al. [61]	Experimental group CRC Control group	Untargeted metabolites identification		Fecal specimen	Distribution (Abundance) of metabolites
		High-performance liquid chromatography/tandem mass spectrometry			
Sinha et al. [62]	Experimental group CRC Control group	Untargeted metabolites identification	Untargeted microbiome identification	Fecal specimen	Distribution (Abundance) of metabolites Distribution of bacterial genera Odds ratio for both microbiota and metabolites
		HPLC-GC/MS-MS	16S rRNA gene sequencing		
Clos-Garcia et al. [63]	Experimental group ADA, CRC Control group	Targeted metabolites identification	Untargeted microbiome identification	Fecal specimen	Distribution (Abundance) of metabolites Distribution of bacterial genera
		UHPLC-MS	16S rRNA gene sequencing		
Tan et al. [66]	Experimental group CRC Control group	Untargeted metabolites identification		Serum specimen	Distribution (Abundance) of metabolites %
		Gas chromatography time-of-flight mass spectrometry (GC−TOFMS)UPLC−QTOFMS			
Flemer et al. [67]	Experimental group ADA CRC Control group	Untargeted microbiome identification		Fecal specimen and mucosa biopsy	Distribution of bacterial species
		16S rRNA gene sequencing			
Zeller et al. [68]	Experimental group ADA CRC Control group	Untargeted microbiome identification		Fecal specimen and mucosa biopsy	Distribution (Abundance) of bacterial genera
		Whole-genome shotgun sequencing of fecal samples) 16S rRNA gene sequencing (DNA from 48 tissue sample pairs (tumor and healthy mucosa) and 129 fecal samples			
Zackular et al. [69]	Experimental group ADA CRC Control group	Untargeted microbiome identification		Fecal specimen	Distribution (Abundance) of bacterial genera
		16S rRNA gene sequencing analysis			
Ohigashi et al. [22]	Experimental group ADA CRC Control group	Targeted metabolites identification	Targeted microbiome identification	Fecal specimen	Distribution (Abundance) of metabolite. Bacterial counts
		Organic acids, identification from stools using high-performance liquid chromatography system.	*Clostridium leptum*, *Bacteroides fragilis*, *Bifidobacterium*, *Atopobium*, *Prevotella*, *Clostridium difficile*, *Clostridium perfringens*, *Lactobacillus casei*, *Lactobacillus gasseri*, *Lactobacillus plantarum*, *Lactobacillus reuteri*, *Lactobacillus ruminis*, *Lactobacillus sakei*, *Lactobacillus brevis*, *Lactobacillus fermentum*, *Lactobacillus fructiborans Enterobacteriaceae*, *Enterococcus*, *Staphylococcus*, *Pseudomonas* using real-time PCR		
Chen et al. [70]	Experimental group ADA Control group	Untargeted metabolites identification	Untargeted microbiome identification	Fecal specimen	Abundance/distribution and concentration of metabolite. Bacterial species distribution/abundance Fold-change in gene expression of bacterial species producing specific metabolites.
		Ion chromatography and ultra-performance liquid chromatography-tandem mass spectrometry (UPLC-MS/MS).	16S rRNA gene sequencing analysis followed by real-time PCR to identify bacteria that produced specific metabolites		
			Targeted microbiome identification		
			Real-time PCR analysis, butyrate-producing bacteria, determined by the presence of the butyryl-coenzyme-A-CoA transferase (*bcoA*) gene, secondary bile acid-producing bacteria, determined by the presence of the Bile acid 7α-dehydroxylation (*baiCD*) gene, conjugated linoleic acid-producing bacteria, determined by the presence of the plasminogen activator inhibitor 1(*pai-1*) gene, plasmid-encoded *cfr* gene (*clbA*) gene and the polypeptide outer membrane usher protein (*afaC*) gene of the *afa-1* operon were used to detect Putative inactive phenolphthiocerol synthesis polyketide synthase type I (*pks1*) bacteria and afa-1 adhesin-expressing diffusely adhering *Escherichia coli* (DAEC), respectively For *F. nucleatum* 16S rRNA gene		
Eklöf et al. [71]	Experimental group ADA/dysplasia CRC Control group	Targeted microbiome identification		Fecal specimen	+/− of *clbA* and *afaC* +, *F. nucleatum* bacteria
		qPCR *clbA* gene colibactin-producing bacteria, diffusely adherent *Escherichia coli* harboring the afa-1 operon, and *F. nucleatum*			
Gao et al. [72]	Experimental group ADA CRC Control group	Untargeted microbiome identification		Fecal specimen	Distribution (Abundance) of bacterial species
		16S rRNA gene sequencing analysis			
Yusuf et al. [73]	Experimental group CRC Control group	Targeted metabolites identification		Fecal specimen	+/− absence of acetate, propionate and butyrate acids
		Acetate, propionate and butyrate acids by gas chromatography			
Weir et al. [74]	Experimental group CRC Control group	Untargeted metabolites identification	Untargeted microbiome identification	Fecal specimen	Distribution (Abundance) of bacterial species, % abundant, fold change Distribution (abundance)
		Gas chromatography-mass spectrometry (GC-MS)	16S rRNA gene sequencing analysis		
		Targeted metabolites identification			
		Gas chromatography-mass spectrometry (GC-MS)			
Yang et al. [75]	Experimental group CRC Control group	Untargeted metabolites identification	Untargeted microbiome identification	Fecal specimen	Distribution (Abundance) of bacterial species,
		Gas chromatography-mass spectrometry (GC-MS)	16S rRNA gene sequencing analysis		

**Table 4:** Included studies identified microbial and metabolites associated with ADA or CRC for diagnostic purposes.

Author	Comparison Group	Bacterial or Metabolite Markers	Performance to Detect ADA or CRC		Identification Technique
			**AUC (CI 95%)**	**Sen/Spec**	
Sun et al. [26]	ADA vs. HC	3 metabolites IPA IALD Indole/Trap ratio	ND	ND	16S rRNA gene sequencing. Ultraperformance liquid chromatography coupled to tandem mass spectrometry.
	ADA vs. HC	4 metabolites Skatole IALD I3CA Indoles	ND	ND	
	CRC vs. HC	10 Bacteria *Bacteroides* *Bacilli* Clostidales_Incertae_Sedis XI Clostridia Fusobacteria Verrucomicrobia Corynebacteriacea Enterobacteriacea 5 metabolites KYN IPA IALD I3CA Indole/Trap ratio	ND	ND	
Kim et al. [50]	AD vs HC	24 metabolites Endocannabinoid N acetyl-cadverine Bilirubin ZZ Lionleoyl ethanolamide Oleoyl ethanolamide Palmitoyl ethanolamide 3-Hydroxy-palmitate Myristoleate Palmitoleate 1-Linoleoyl-GPE 1-Palmitioyl -GPE Secondary bile acid 3b-Hydroxy-5-cholenoic acid Deoxycholate Polyunsaturated fatty acid Docosahexaenoate Docosapentaenoate Hexadecadienoate Sphingolipid N-palmitoyl-saphinganine Hexadecasphinganine Sphinganine Piperine 3,7-Dimethyl-urate	ND	ND	UPLC-MS/MS platform
	CRC vs. HC	8 metabolites Polyunsaturated fatty acid Docosahexaenoate Docosapentaenoate Hexadecadienoate Sphingolipid N-palmitoyl-saphinganine Hexadecasphinganine Sphinganine Piperine 3,7-Dimethyl-urate	ND	ND	
Nugent et al. [52]	ADA vs. HC	23 metabolites Galactose, 13,14-dihydro-15-keto-PGE2, 5-oxoproline, 2,4-diaminobutyric acid, Pentadecanoic acid, 5-hydroxyindoleacetic acid, Phosphoric acid, 2-aminoethanol, Dihydroceramide, Ornithine, linoleic acid, Petroselinic acid, LysoPC (18:2(9Z,12Z)), Myo-inositol, Diketogulonic acid, Prostaglandin E2, Methionine, 2-aminobutyric acid, Oleamide, Glycine, Maltitol, 2-phenylglycine, 2-phenylacetamide, N6-acetyl-L-lysine	ND	ND	Liquid chromatography and gas chromatography time of flight mass spectrometry
Chang et al. [53]	CRC vs. HC	18 bacteria *Parvimonas micra* *Fusobacterium nucleatum* *Clostridium saccharoperbutylacetonicum* *Clostridium beijerinckii* *Eubacterium celluloslvens* *Lachnoclostridium phytofermentans* *Clostridium butyricum* *Herbiirix luporum* *Balcillus cereus* *Blautia* sp. *SCOSB48* *Anaerobutyrucium hallii* *Lachnospiraceae bacterium Choco86* *Eubacterium eligens* *Blautia hansenii* *Longibaculum SPKGMB06250* *Clostridum sporogenes* *Faecalibacterium prausnitizi* *Anaerostipes hardus*	ND	ND	Whole-genome shotgun sequencing
Guertin et al. [54]	CRC vs. HC	1 metabolite Serum choline	ND	ND	Liquid chromatography (LC) tandem mass spectrometry (MS)
Kim et al. [56]	CRC vs. HC	2 Bacteria *Solanum melongena*, *Collinsella*	95%	ND	16S rRNA gene sequencing Gas chromatography-time-of-flight mass spectrometry
		2 metabolites Leucine and Oxalic acid	92%	ND	
		Both bacteria+ metabolites *Solanum melongena*, *Collinsella*, Leucine and Oxalic acid	100%	ND	
Song et al. [57]	CRC vs. HC	4 metabolites Monounsaturated fatty acid (MUFAs), Oleic acid, ω-6-polyunsaturated fatty acids (ω-6 PUFAs), and Linoleic acid	ND	ND	Gas chromatography-mass Spectrometry
Genua et al. [58]	ADA vs. CRC	1 metabolite 2-MethylButyric acid			Gas chromatography
	CRC vs. HC	4 metabolites Acetic acid, Propnic acid, i-Valeric, and Valeric acid	ND	ND	
D’asheesh et al. [59]	CRC vs. HC	3 Bacteria *Lactobacillus acidophilus*, *Lactobacillus palntarom*, and *Enterococcus faecalis*	ND	ND	Real-time PCR
Coker et al. [60]	ADA vs. CRC	6 bacteria *Roseburia inulinivorans* *Xanthmonas perforans* *Fusobacterium nucleatum* *Eiknella corrodens* *Parvimonas micra* *Peptostreptococcus anaerobius* 11 metabolites 2-Hydroxy butyric acid Gamma Aminobutyric acid L-alanine L-Aspartic acid Norvaline Orinthine Oxoadipic acid Oxoglutaric acid Palmitoleic acid Pimelic acid	Only bacteria 94.17% (91.5–96.83)	ND	Whole-genome shotgun sequencing Gas chromatography coupled to time-of-flight mass Spectrometer (GC-TOFMS)
	ADA vs. HC	14 bacteria *Roseburia inulinivorans* *Xanthmonas gardneri* *Fusobacterium nucleatum* *Prevotella intermedia* *Peptostreptococcus stomatis* *Sutterella parviruba* 4 metabolites Alpha-Linoleici acid L-Homoserine Phenylacetic acid Phenyllactic ac	Only bacteria 87.59% (83.58, 91.6%)	ND	
	CRC vs. HC	14 bacteria *Eubacteria cellulosolvens* *Lachinospiraceae_bacterium-3-1-57FAA-CT1* *Clostridium bolteae* *Streptococcus tigurinus* *Xanthmonas gardneri* *Eikenella corrodens* *Oscillibacter valericigens* *Actinomyces viscosus* *Synergistes_sp_1_syn1* *Clostridium symbiosum* *Prevotella intermedia* *Slackia exigua* *Prevotella nigrescens* *Porphymonas gingivalis* 2 metabolites L-Asparagine Phenyllactic acid	Both 14 bacteria and 2 metabolites 93.7% (91.07, 96.42%)	ND	
Goedert et al. [61]	CRC vs. HC	10 metabolites 3-Dehydrocarnitine, p aminobenzoate (PABA) α-Tocopherol, γ-Tocopherol, Pterin, N-2-Furoyl-glycine, p-Hydroxybenzaldehyde, Sitostanol, Conjugated linoleate-18-2N7, Palmitoyl-sphingomyelin, Mandelate	77%	ND	High-performance liquid chromatography/tandem mass spectrometry
Sinha et al. [62]	CRC vs. HC	4 Bacteria *Fusobacterium*, *g-Porphyromonas*, Clostridia, Lachnospiraceae 5 metabolites p-hydroxy-benzaldehyde, Palmitoyl-sphin-gomyelin p-aminobenzoate, Conjugated linoleate, and Mandelate	ND	ND	16S rRNA gene sequencing HPLC-GC/MS-MS
Clos-Garcia et al. [63]	ADA vs. H	1 metabolite Triacylglycerol	ND	ND	16S rRNA gene sequencing UHPLC-MS
	ADA vs. CRC	4 Bacteria *Streptococcus* *Parvvimonas* *Coriobacteriaceae* *Adlercreutzia* 3 metabolites cholesteryl esters, sphingolipids, Glycerophospatidylcholine	ND	ND	
	CRC vs. HC	7 Bacteria *Fusobacterium*, *Streptococcus*, *Parvimonas*, *Coprococcus*, *Blatia*, *Clostridum*, *Staphylococcus* 3 metabolites Cholesteryl esters, sphingolipids, Glycerophospatidylcholine	ND	ND	
Tan et al. [66]	CRC vs. HC	72 metabolites This involved the following categories: Tricarboxylic acid (TCA) cycle, urea cycle, glutamine, fatty acids, and gut flora metabolism Tan et al. [66]	ND	ND	Gas chromatography time-of-flight mass spectrometry (GC−TOFMS) UPLC−QTOFMS
Flemer et al. [67]	CRC vs. HC	6 Bacteria *Bacteroides* *Roseburia* *Ruminococcus* *Oscillibacter* *Lachinospiraceae incertae* *Coporoccus*	87%	ND	16S rRNA gene sequencing
Zeller et al. [68]	CRC vs. HC	2 Bacteria *Fusobacterium nucleatum* subsp. vincentii and *Fusobacterium nucleatum* subsp. animalis	85% (84–87%)	ND	Whole-genome shotgun sequencing/16S rRNA gene sequencing
Zackular et al. [69]	ADA vs. HC	6 Bacteria *Fusobacterium*, *Porphyromonas*, *Lachnospiraceae*, Enterobacteriaceae, *Bacteroides*, Lachnospiraceae Clostridiales	79.8% (68.7–90.8%)	ND	16S rRNA gene sequencing
	ADA vs. CRC	4 Bacteria *Fusobacterium*, *Porphyromonas*, *Parasutterella* *Pacscolarctobacterium*	82.3% (72.2–92.3%)	ND	
	CRC vs. HC	6 Bacteria *Fusobacterium*, *Porphyromonas*, *Lachnospiraceae*, Enterobacteriaceae, *Bacteroides*, Lachnospiraceae and Clostridiales	83.9% (74–93.8%)	ND	
Ohigashi et al. [22]	ADA vs. CRC	3 Bacteria *Clostridium leptum*, *Bacteroides fragilis*, *Staphylococc*	ND	ND	Real-time PCR Liquid chromatography system
	CRC vs. HC	7 Bacteria *C. coccoides*, *C. leptum*, *B. fragilis*, *Bifidobacterium*, *Atopobium*, Enterobacteriaceae, *Staphylococcu* 4 Metabolites Acetic acid, Propionic acid, Butyric acid, and Valeric acid	ND	ND	
Chen et al. [70]	ADA vs. HC	1 Bacterium *Bacteroidete* 3 Metabolites Acetic acid, butyric acid, and *t10*, *c12-CLA*	Both 90% (70–90%)	ND	16S rRNA gene sequencing analysis followed by real-time PCR. Ion chromatography and ultra-performance liquid chromatography-tandem mass spectrometry (UPLC-MS/MS).
Eklöf et al. [71]	ADA/dysplasia vs. CRC	1 Bacterium *F. nucleatum*	73.7%	84.6% and 63.1%	Real-time PCR
Gao et al. [72]	ADA vs. HC and CRC vs. HC	18 Bacteria *Rhodococcus*, *Anaerostipes*, *Escherichia_Shigella*, *Akkermansia*, *Gemella*, *Clostridium_XVIII*, *Alkaliphilus Paenibacillus*, *Enterococcus*, *Fusobacterium*, *Fusicatenibacter*, *Blautia Porphyromonas*, *Faecalibacterium*, *Parvimonas*, *Peptostreptococcus*, *Clostridium_IV Bacillus*	ADA vs. HC 61.6% (52–71%) CRC vs. HC 85.8% (78–93%)	ADA vs. HC 83.6% and 39% CRC vs. HC 66.7% and 98%	16S rRNA gene sequencing
Yusuf et al. [73]	CRC vs. HC	3 Metabolites Acetate, propionate and butyrate acids	ND	ND	Gas Chromatography
Weir et al. [74]	CRC vs. HC	18 Bacteria *Bacteroides finegoldii*, *Bacteroides intestinalis*, *Prevotella copri*, *Prevotella oris*, *Ruminococcus obeum*, *Dorea formicigenerans*, *Lachnobacterium bovis*, *Lachnospira pectinoschiza*, *Pseudobutyrivibrio ruminis*, *Bacteroides capillosus*, *Ruminococcus albus*, *Dialister invisus*, *Dialister pneumosintes*, *Megamonas hypermegale*, *Acidaminobacter unclassified*, *Phascolarctobacterium unclassified*, *Citrobacter farmer*, *Akkermansia muciniphila*,	ND	ND	16S rRNA gene sequencing analysis Gas chromatography—mass spectrometry (GC-MS)
		20 Metabolites Alanine, Glutamate, Glycine, Aspartic acid, Leucine, Lysine, Proline, Threonine, valine, Phenylalanine, Benzeneacetic acid, Propionic acid, pantothenic acid, Cholesterol derivatives, Oleic acid, Linoleic acid, Elaidic acid, Glycerol, Monooleoylglycerol, Ursodeoxycholic acid	ND	ND	
Yang et al. [75]	CRC vs. HC	13 Bacteria *Escherichia-Shigella*, *Parvimonas*, *Fusobacterium*, *CFT112H7_norank*, *Porphyromonas. Firmicutes*, *Clostridiales*, *Clostridia*, *Lachnospiraceae*, *Ruminococcaceae*, *Selenomonadales*, *Negativicutes*, and *Faecalibacterium*	ND	ND	Gas chromatography—mass spectrometry (GC-MS) 16S rRNA gene sequencing analysis
		2 metabolites Cadaverine putrescine	Only metabolites, each one alone: 74% 67.2	ND	

**Table 5:** Quality appraisal of the included studies.

Author	Recruitment/5	Examiner/2	Methodology/5	Outcomes/2	Missing Data/7	Statistical Analysis/5	Results/2	Overall Score/28	Overall Score 100%
Zhen Sun et al. [26]	4	0	3	2	7	3	2	21	77.7
Kim et al. [50]	4	0	5	2	7	5	2	25	92.5
Nugent et al. [52]	4	0	2	2	7	2	2	19	70.3
Chang et al. [53]	0	0	1	2	7	3	1	14	51.8
Guertin et al. [54]	1	2	5	2	7	5	2	24	88.8
Kim et al. [56]	4	0	4	2	7	5	2	24	88.8
Song et al. [57]	4	0	3	2	7	3	1	20	74.1
Genua et al. [58]	2	0	5	2	6	5	1	20	74.1
D’asheesh et al. [59]	3	0	3	2	4	2	0	14	51.8
Coker et al. [60]	4	0	5	2	7	5	2	25	92.5
Goedert et al. [61]	2	1	2	2	6	2	1	16	59.3
Sinha et al. [62]	2	0	5	2	7	5	2	23	85.2
Clos-Garcia et al. [63]	1	0	5	2	7	5	2	23	81.1
Tan et al. [66]	4	0	5	2	7	3	1	22	81.1
Flemer et al. [67]	4	0	5	2	7	5	2	25	92.6
Zeller et al. [68]	4	0	5	2	7	5	2	25	92.6
Zackular et al. [69]	4	0	5	1	6	3	2	21	77.8
Ohigashi et al. [22]	4	0	3	2	6	1	1	17	62.9
Chen et al. [70]	4	0	3	2	6	4	1	20	74.1
Eklöf et al. [71]	2	0	3	2	6	3	1	17	62.9
Gao et al. [72]	3	0	2	2	7	2	1	17	62.9
Yusuf et al. [73]	3	0	1	2	6	2	1	15	55.5
Weir et al. [74]	4	0	2	2	7	2	1	18	66.7
Yang et al. [75]	4	0	5	2	7	3	2	23	85.2

**Table 6:** Levels of evidence for summary statements for each intervention.

**a. Untargeted Microbiome Identification**										
**Study (Appraisal Quality)**	**Increased in ADA vs. HC**			**Increased in CRC vs. ADA**		**Increased in CRC vs. HC**				
Nugent et al. [52] 66.6% (L)	*Bifidobacterium* sp. *Eubacteria*									
Chang et al. [53] 51.8% (L)						*Streptococcus gallolyticus*, *Haemophillus parainfluenza*, *Dialister* sp. *Marseille-P5638*, *Ruthenibacterium lactatiformans*				
Kim et al. [56] 88.8% (H)						*Bifidobacterium*, *Collinsella*, *Blautia*, *Lachnoclostridium* Lachnospiraceae, *Dorea* *Eubacterium coprostanoligenes group* Ruminococcaceae-*Ruminococcus* *Faecalibacterium*, *Subdoligranulum* *Catenibacterium*, *Parvimonas* *Ruminiclostridium*, *Enterobacter* *Diaphorobacter*				
Sinha et al. [62] 85.2% (H)						*Fusobacterium*, *Porphyromonas* *Clostridia*, Lachnospiraceae				
Flemer et al. [67] 92.6% (H)						*Bacteroides*, *Roseburia* *Ruminococcus*, *Oscillibacter* *Porphyromonas*, *Peptostreptococcus*, *Parvimonas*, *Fusobacterium*				
Zeller et al. [68] 92.6% (H)				*Fusobacterium nucleatum *subsp.* vincentii* *Fusobacterium nucleatum *subsp.* Animalis* *Fusobacterium nucleatum *subsp.* nucleatum* *Fusobacterium nucleatum *subsp.* polymorphum* *Porphyromonas asaccharolytica* *Prevotella nigrescens* *Peptostreptococcus stomatis* *Parvimonas* sp. *Parvimonas micra* *Olsenella uli* *Parvimonas* sp. *Streptococcus anginosus*		*Fusobacterium nucleatum *subsp.* vincentii* *Fusobacterium nucleatum *subsp.* Animalis* *Fusobacterium nucleatum *subsp.* nucleatum* *Pseudoflavonifractor capillosus* *Fusobacterium nucleatum *subsp.* polymorphum* *Porphyromonas asaccharolytica* *Ruminococcaceae bacterium* *Prevotella nigrescens* *Peptostreptococcus stomatis* *Leptotrichia hofstadii* *Parvimonas* sp. *Parvimonas micra* *Bacteroides fragilis* *Bilophila wadsworthia* *Neisseria* sp. *Campylobacter rectus* *Selenomonas sputigena* *Leptotrichia buccalis* *Clostridium hylemonae* *Clostridium symbiosum*				
Zackular et al. [69] 77.8% (H)	Ruminococcaceae *Clostridium* *Pseudomonas* Porphyromonadaceae			*Fusobacterium* *Bacteroides* *Phascolarctobacterium* *Porphyromonas*		*Fusobacterium* *Porphyromonas* *Lachnospiraceae* Enterobacteriaceae				
Chen et al. [70] 74.1 (H)	*Bacteroides* *Escherichia* *Faecalibacterium* *Citrobacter*									
Gao et al. [72] 62.9% (L)	*Bacillus cereus* *Bacillus thuringiensis* *Bacillus amyloliquefaciens* *Cronobacter sakazakii*			*Alcanivorax hongdengensis* *Burkholderia mallei* *Clostridium ramosum* *Coprobacillus* sp. *Fusobacterium* sp.		*Streptococcus intermedius* *Peptostreptococcus stomatis* *Parvimonas micra* *F. nucleatum*				
Weir et al. [74] 66.7% (L)						*Acidaminobacter Citrobacter farmer* *Akkermansia muciniphila*				
Yang et al. [75] 85.2% (H)						Enterobacteriaceae *Fusobacterium*				
Increased in ADA vs. HC										
Overlapping microbial markers	No common microbial markers 4 studies [52,69,70,72]									
Level of evidence	Conflicting									
Increased in CRC vs. ADA										
Overlapping microbial markers	*Fusobacterium* sp. 3 studies [68,69,72]					*Porphyromonas* *2 studies* [68,69]				
Level of evidence	Strong					Strong				
Increased in CRC vs. HC										
Overlapping microbial markers	Lachnospiraceae-*Lachnoclostridium* 3 studies [56,62,69]	Ruminococcaceae-*Ruminococcus* 4 studies [56,62,67,68]	*Parvimonas* *Parvimonas micra* 4 studies [56,67,68,72]	Enterobacteriaceae 2 studies [69,75]	*Fusobacterium* sp. 5 studies [62,67,68,69,75]	*Bacteroides* 2 studies [67,68]	*Peptostreptococcus* sp. 2 studies [67,72]	*Clostridia* sp. *C. hylemonae* *C. symbiosum* 2 studies [62,68]	*Porphyromonas* *4 studies* [62,67,68,69]	*Streptococcus* sp. *S. gallolyticus*, *S. intermedius* 2 studies [53,72]
Level of evidence	Strong	Strong	Strong	Strong	Strong	Strong	Strong	Strong	Strong	Limited
**b. Targeted microbiome identification**										
**Study (Appraisal quality)**			**Increased in ADA vs. HC**			**Increased in CRC vs. ADA**			**Increased in CRC vs. HC**	
D’asheesh et al. [59] 51.8 (L)			*Bifidobacterium *sp.* Eubacteria*						*Enterococcus faecalis*	
Clos-Garcia et al. [63] 81.1% (H)						*Staphylococcus and Parvimonas*			*Fusobacterium*, *Staphylococcus and Parvimonas*	
Ohigashi et al. [22] 62.9% (L)									*C. difficile* *C. perfringens*, *Pseudomonas* *^,1^	
Eklöf et al. [71] 62.92% (L)									*F. nucleatum*	
**Increased in ADA vs. HC**										
Overlapping microbial markers			Only one study was reported. [12]							
Level of evidence			NO							
**Increased in CRC vs. ADA**										
Overlapping microbial markers			Only one study was reported. [63]							
Level of evidence			NO							
**Increased in CRC vs. HC**										
Overlapping microbial markers			Fusobacterium sp. 2 studies [63,71]							
Level of evidence			Moderate							
**c. Untargeted Metabolites Identification**										
**Study (Appraisal quality)**	**Increased in ADA vs. HC**				**Increased in CRC vs. HC**					
Kim et al. [56] 92.5% (H)	Endocannabinoid N acetyl-cadverine Bilirubin ZZ Lionleoyl ethanolamide Oleoyl ethanolamide Palmitoyl ethanolamide 3-Hydroxy-palmitate Myristoleate Palmitoleate 1-Linoleoyl-GPE 1-Palmitioyl -GPE		Polyunsaturated fatty acid Docosahexaenoate Docosapentaenoate Hexadecadienoate							
	Secondary bile acid 3b-Hydroxy-5-cholenoic acid Deoxycholate		Sphingolipid N-palmitoyl-saphinganine Hexadecasphinganine Sphinganine Piperine 3,7-Dimethyl-urate							
Nugent et al. [52] 66.7% (L)	The inflammatory metabolite prostaglandin E2									
Kim et al. [50] 88.8% (H)	Aminoacids Leucine Isoleucine Alanine Lysine Tyramine Aminoisobutyric acid		Amino alcohol Ethanolamine Aromatic alcohol Phenol							
	Carboxylic acid Furoic acid Succinic acid Oxalic acid		Fatty acid Butanoic acid Hexanoic acid Palmitic acid Oleic acid							
Godert et al. [61] 59.3% (L)					Heme-related molecules Heme Z-18565 X_19549			Cofactors. and vitamin α-Tocopherol γ-Tocopherol Pterin		
					Xenobiotics 4-Acetamidophenol 2-Hydroxyacetaminophen sulfate 3-Cystein-S-YL-acetaminophen p-Acetamidophenylglucuronide Para-aminobenzoic acid (PABA) N-2-Furoyl-glycine Sitostanol p-Hydroxybenzaldehyde Mandelate			Peptides/Aminoacids Histidine Cis-Urocanate Tryptophyl-glycine Leucyl-tryptophan Alanyl-histidine Histidyl-glycine Tyrosylglutamine Histidyl-alanine Valyl-aspartate Pyro-glutamyl-glycine Alanyl-leucine Alanyl-tryptophan Histidylphenylalanine Leucyl-glutamate Leucyl-serine α-Glutamyl-valine Prolyl-alanine Valyl-histidine		
					Lipids Palmitoyl-sphingomyelin Conjugated linoleate-18-2N7 3-Dehydrocarnitine					
Shina et al. [62] 85.5% (H)					Palmitoyl_Sphingomyelin p_Hydroxybenzaldhyde					
Tan et al. [66] 81.1% (H)					Fatty acid metabolism β-hydroxybutyrate betaine Glycerol Oleamide Oleic acid Erythrotetrofuranose Carnitine (18:1) Linolic acid Acetyl carnitine Elaidic acid 3-oxodecanoic acid Palmitic acid		valine, leucine, and isoleucine degradation Allisoleucine		Arginine and proline metabolism Creatinine	
					Purine nucleotide synthetics Xanthosine		Cystine & methionine metabolism Cystine		Carbohydrate metabolism Threitol	
					Phospholipid metabolism Sphinganine CPA(18:0/0:0)		Glutathione metabolism 2-hydroxybutyric acid 2-aminobutanoic acid TCA cycle Pyruvate Vitamin B6 metabolism Glycolaldehyde		Others Tetrahydrogestrinone Allyl isothiocyanate Proline	
Weir et al. [74] 66.7% (L)					Aminoacids Alanine Glutmate Glycine Aspartic acid Leucine Lysine Proline Serine Threonine Valine Phenylalanine			Carboxylic acids Beneneacetic acid Propionic acid Mysteric acid Pantothenic acid		
					Steroids Cholesterol derivative					
Yang et al. [75] 85.2% (H)					4-Methylvaleric acid 9-(2-Carboxyethyl)-2,2,4,4-tetramethyl-1,2,3,4-tetrahydro-gamma-carboline Adenosine Butanoic acid d-2-Aminobutyric acid DL-Ornithine D-Proline, n-propoxycarbonyl-, hexadecyl ester Heptanedioic acid Heptanoic acid Hexane, 2,5-dimethyl L-5-Hydroxytryptophan L-Lysine L-Tryptophan L-Norleucine L-Norvaline Pentanoic acid N-Acetyl-D-glucosamine Cadaverine					
**Increased in ADA vs. HC**										
Overlapping metabolite markers	No common metabolites 5 studies [50,52,56,74,75]									
Level of evidence	Conflicting									
**Increased in CRC vs. HC**										
Overlapping metabolite markers	Palmitoyl-sphingomyelin 2 studies [61,62]				Proline 2 studies [66,74]					
Level of evidence	Moderate				Moderate					
**d. Targeted metabolites identification**										
**Study (Appraisal Quality)**	**Increased in ADA vs. HC**			**Increased in CRC vs. ADA**			**Increased in CRC vs. HC**			
Zhen Sun et al. [26] 77.7% (H)	Kynurenin(KYN) Indole-3-aldehyde (IALD) and Indole-3-carboxylic acid (I3CA) The ratio of KYN to Trp (KYN/Trp ratio)						Kynurenin(KYN) Indole-3-aldehyde (IALD) and Indole-3-carboxylic acid (I3CA) The ratio of KYN to Trp (KYN/Trp ratio)			
Guertin et al. [54] 88.8% (H)							Serum choline			
Song et al. [57] 74.1% (L)							Monounsaturated fatty acids (MUFAs) C18:1ω-9 Oleic acid ω-6 polyunsaturated fatty acids (PUFAs) C18:2ω-6 Linoleic acid			
Genua et al. [58] 74.1% (L)							2-MethylButyric Acid Acetic Acid Propionic acids			
Coker et al. [60] 92.5% (H)			Phenyllactic acid, Phenylacetic acid, L-Phenylalanine, L-Valine, L-Alpha-aminobutyric acid, L-Proline, L-Alanine Oxoglutaric acid, L-Isoleucine, Gamma-Aminobutyric acid, L-Leucine, Glycine, L-Methionine, L-Tyrosine, L-Aspartic acid, Butyric acid, Glutathione, Succinic acid, 2-Hydroxybutyric acid, Malic acid, 3-Aminoisobutanoic acid, Ornithine, Beta-Alanine, Myristic acid, Oxoadipic acid, Alpha-Linolenic acid, L-Serine, Nicotinic acid, Linoleic acid, Pelargonic acid, Pyroglutamic acid, Glutaric acid, Hexanoic acid, L-Homoserine, 5-Dodecenoic acid, Pimelic acid				L-alanine, glycine L-proline L-aspartic acid L-valine L-leucine L-serine myristic acid phenyl lactic acid oxoglutaric acid L-phenylalanine L-alpha-aminobutyric acid phenylacetic acid palmitoleic acid 3-aminoisobutanoic acid norvaline			
Ohigashi et al. [22] 62.9% (M)							Succinic acid			
Yusuf et al. [73] 55.5% (M)							The opposite decrease in Acetate Propionate butyrate acids			
**Increased in ADA vs. HC**										
Overlapping microbial markers	Only one study [19]									
Level of evidence	NO									
**Increased in CRC vs. ADA**										
Overlapping microbial markers	Only one study [60]									
Level of evidence	NO									
**Increased in CRC vs. HC**										
Overlapping microbial markers	No common metabolites 6 studies [26,54,57,60,73,75]									
Level of evidence	Conflicting									
**e. Untargeted microbial markers for tumor stages and locations**										
**Study (Appraisal Quality)**	**Microbial Markers in CRC Early Stage I**	**Microbial Markers in CRC III Stage**		**Microbial Markers in CRC IV, Late Stage**	**Microbial Markers in Distal Cancers vs. Proximal Cancers**		**Microbial Markers in Rectal vs. Proximal Cancers**		**Microbial Markers in Proximal Cancer**	
Flemer et al. [67] 92.6% (H)					Alistipes Akkermansia Halomonas Shewanella		Alistipes Akkermansia Halomonas Shewanella		Faecalibacterium Blautia Clostridium	
Gao et al. [72] 62.9% (M)	Escherichia/Shigella	Bacteroides		Saccharibacteria incertaesedis	Escherichia/Shigella					
**Microbial markers in CRC early stage I**										
Overlapping microbial markers	Only one study reported. [72]									
Level of evidence	NO									
**Microbial markers in CRC III stage**										
Overlapping microbial markers	Only one study reported. [72]									
Level of evidence	NO									
**Microbial markers in CRC IV, late-stage**										
Overlapping microbial markers	Only one study reported. [72]									
Level of evidence	NO									
**Microbial markers in distal cancers vs. proximal cancers**										
Overlapping microbial markers	No common metabolites Two studies [67,72]									
Level of evidence	Conflicting									
**Microbial markers in rectal vs. proximal cancers**										
Overlapping microbial markers	Only one study reported. [67]									
Level of evidence	NO									
**Microbial markers in proximal cancer**										
Overlapping microbial markers	Only one study reported. [67]									
Level of evidence	NO									
**f. Targeted microbial markers for tumor stages and locations**										
**Study (Appraisal Quality)**	**Microbial Markers in CRC IV, Late Stage**					**Microbial Markers on Right Side**				
Clos-Garcia et al. [63] 81.1% (H)	Bulleidia Fusobacterium Butyrivibrio Peptostreptococcus Staphylococcus Parvimonas Selenomonas									
Ohigashi et al. [22] 62.9% (M)						Clostridium perfringens				
**Microbial markers in CRC IV, late-stage**										
Overlapping microbial markers	Only one study reported. [63]									
Level of evidence	NO									
**Microbial markers on right side**										
Overlapping microbial markers	Only one study reported. [22]									
Level of evidence	NO									
**g. Untargeted metabolite markers for tumor stage and location**										
**Study (Appraisal Quality)**	**Microbial Markers in CRC Late Stage IV vs. Early Stage I**									
Tan et al. [66] 81.1% (H)	Beta hydroxybuturate									
**Microbial markers in CRC late stage IV vs. early stage I**										
Overlapping microbial markers	Only one study reported. [66]									
Level of evidence	NO									

* ^1^ healthy control included adenoma and non-adenoma participants.

## Data Availability

Not applicable.

## Supplementary Materials

The following supporting information can be downloaded at: https://www.mdpi.com/article/10.3390/microorganisms11082037/s1, Supplementary material of this systematic review can be found in the online version. Table S1. Extraction form, Table S2. Appraisal quality form.
