# Supplementary material for: Gut Microbial and Associated Metabolite Markers for Colorectal Cancer Diagnosis
Source: Microorganisms. 2023 Aug 8;11(8):2037. doi: 10.3390/microorganisms11082037 (PMC10457972; doi:10.3390/microorganisms11082037)
Supplement: Supplementary file 1 [file microorganisms-11-02037-s001.zip › microorganisms-2478040-supplementary.pdf]

Table S1 Appendix: SYSTEMATIC LITERATURE REVIEW DATA EXTRACTION FORM

Write here a reviewer name:

Click here to enter a date.

Study description

ID No.	

STUDY SELECTION CRITERIA

Inclusion	Exclusion
☐ English	☐ Other languages
☐ Gut microbiome and metabolites	☐ Conference Proceedings
☐ Original study	☐ Case Series < 10 Subjects
☐ Colorectal cancer	☐ Case Studies
☐ Full text available	☐ Systematic Reviews
	☐ Narrative review
	☐ Protocols

DECISION: ☐ Included ☐ Excluded ☐ Unclear

Study goals, type and timeline

Goal of the study	
Type of the study	
Timeline of study	Click to select the timeline of the study

Subject selection criteria.

Inclusion Criteria?	☐ Not reported ☐ Unclear ☐ Yes, Specify:
Exclusion Criteria?	☐ Not reported ☐ Unclear ☐ Yes, Specify:
Subject recruitment	☐ Random ☐ Consecutive ☐ Volunteers ☐ Purposeful.

Groups (definition) or define reported group and subgroups

Group name:			
No of participants			
Age			
Diagnosis			
Gender	# of male:<br># of female:	# of male:<br># of female:	# of male:<br># of female:

List of measurements outcomes (for every subgroup/time-point/measure)
Gut microbiome species for adenoma or carcinoma in colorectal cancer patients compared to healthy group
Gut metabolite types for adenoma or carcinoma in colorectal cancer patients compared to healthy group
Other, specify:

Results of statistical test of correlations between gut microbiome and adenoma or carcinoma; gut metabolites and adenoma or carcinoma; and gut microbiome species and gut metabolites:
Name of the association test used:
Variables tested:
Group tested:
Association estimate (correlation coefficient, regression equations)
P-values:
:

Table S2 Appendix: Appraisal Form

1 **Title: Alteration of fecal tryptophan metabolism correlates with shifted microbiota and may be involved in pathogenesis of colorectal cancer**

Criterion	Yes	No	NA	Comments
**Subjects recruitment**				
Are the characteristics of the participants included in the study clearly described?	☒	☐	☐	
Were the demographic characteristics of the sample reported for each group analyzed?	☒	☐	☐	
Were the subjects asked to participate in the study representative of the entire population from which they were recruited?	☒	☐	☐	
Were those subjects who were prepared to participate representative of the entire population from which they were recruited?	☒	☐	☐	
**Examiners**				
Was/were the person(s) who is doing the experiment blinded to the study groups?	☐	☒	☐	
Was/were the person(s) who is doing the experiment blinded to quality control runs?	☐	☒	☐	
**Methodology**				
Are the exposures/interventions of interest clearly described?	☒	☐	☐	
Was the sample size included in the analysis adequate?	☒	☐	☐	
Is replication of the assessment procedure possible? (description sufficiently detailed)	☒	☐	☐	

Criterion	Yes	No	NA	Comments
Are the distributions of principal confounders in each group of subjects to be compared clearly described?	☐	☒	☐	
Was there an adequate adjustment for confounding in the analyses from which the main findings were drawn?	☐	☒	☐	
**Outcomes**				
Is the hypothesis/aim/objective of the study clearly described? Must be explicit	☒	☐	☐	
Validity reported for the main outcome measure	☒	☐	☐	
**Handling Missing Data (Concurrent and Criterion Validity)**				
Compliance acceptable in all groups (80% acceptable)	☒	☐	☐	
Was the percentage of missing items given	☒	☐	☐	
Withdrawal/dropouts rate described and acceptable	☒	☐	☐	
Have the characteristics of participants lost to follow-up been described?	☒	☐	☐	
Was compliance with the intervention/s reliable?	☒	☐	☐	
Was there a description of how missing items were handled?	☒	☐	☐	
Were hypotheses regarding correlations or mean differences formulated a priori (i.e. before data collection)?	☒	☐	☐	
**Statistical Analysis**				
Have actual probability values been reported (e.g. 0.035 rather than <0.05) for the main outcomes except where the probability value is less than 0.001?	☒	☐	☐	
Sample size described for each group	☒	☐	☐	
Were design and statistical methods adequate for the hypotheses to be tested?	☐	☒	☐	

Criterion	Yes	No	NA	Comments
Has confidence interval for change in outcomes from before to after intervention been reported?	☐	☒	☐	
Have effect sizes for outcomes been reported or can be computed by the reviewer?	☒	☐	☐	
**Results**				
Are the main findings of the study clearly described?	☒	☐	☐	
Have all important adverse events that may be a consequence of the intervention been reported?	☒	☐	☐	

2. Title: Fecal Metabolomic Signatures in Colorectal Adenoma Patients Are Associated with Gut Microbiota and Early Events of Colorectal Cancer Pathogenesis

Criterion	Yes	No	NA	Comments
**Subjects recruitment**				
Are the characteristics of the participants included in the study clearly described?	☒	☐	☐	
Were the demographic characteristics of the sample reported for each group analyzed?	☒	☐	☐	
Were the subjects asked to participate in the study representative of the entire population from which they were recruited?	☒	☐	☐	
Were those subjects who were prepared to participate representative of the entire population from which they were recruited?	☒	☐	☐	
**Examiners**				
Was/were the person(s) who is doing the experiment blinded to the study groups?	☐	☒	☐	
Was/were the person(s) who is doing the experiment blinded to quality control runs?	☐	☒	☐	
**Methodology**				
Are the exposures/interventions of interest clearly described?	☒	☐	☐	
Was the sample size included in the analysis adequate?	☒	☐	☐	
Is replication of the assessment procedure possible? (description sufficiently detailed)	☒	☐	☐	

Criterion	Yes	No	NA	Comments
Are the distributions of principal confounders in each group of subjects to be compared clearly described?	☒	☐	☐	
Was there an adequate adjustment for confounding in the analyses from which the main findings were drawn?	☒	☐	☐	
**Outcomes**				
Is the hypothesis/aim/objective of the study clearly described? Must be explicit	☒	☐	☐	
Validity reported for the main outcome measure	☒	☐	☐	
**Handling Missing Data (Concurrent and Criterion Validity)**				
Compliance acceptable in all groups (80% acceptable)	☒	☐	☐	
Was the percentage of missing items given?	☒	☐	☐	
Withdrawal/dropouts rate described and acceptable	☒	☐	☐	
Have the characteristics of participants lost to follow-up been described?	☒	☐	☐	
Was compliance with the intervention/s reliable?	☒	☐	☐	
Was there a description of how missing items were handled?	☒	☐	☐	
Were hypotheses regarding correlations or mean differences formulated a priori (i.e. before data collection)?	☒	☐	☐	
**Statistical Analysis**				
Have actual probability values been reported (e.g. 0.035 rather than <0.05) for the main outcomes except where the probability value is less than 0.001?	☒	☐	☐	
Sample size described for each group	☒	☐	☐	
Were design and statistical methods adequate for the hypotheses to be tested?	☒	☐	☐	

Criterion	Yes	No	NA	Comments
Has confidence interval for pre- and post-intervention or change in outcomes from before to after intervention been reported?	☒	☐	☐	
Have effect sizes for outcomes been reported or can be computed by the reviewer?	☒	☐	☐	
**Results**				
Are the main findings of the study clearly described?	☒	☐	☐	
Have all important adverse events that may be a consequence of the intervention been reported?	☒	☐	☐	

3. Title: Altered Tissue Metabolites Correlate with Microbial Dysbiosis in Colorectal Adenomas

Criterion	Yes	No	NA	Comments
**Subjects recruitment**				
Are the characteristics of the participants included in the study clearly described?	☒	☐	☐	
Were the demographic characteristics of the sample reported for each group analyzed?	☒	☐	☐	
Were the subjects asked to participate in the study representative of the entire population from which they were recruited?	☒	☐	☐	
Were those subjects who were prepared to participate representative of the entire population from which they were recruited?	☒	☐	☐	
**Examiners**				
Was/were the person(s) who is doing the experiment blinded to the study groups?	☐	☒	☐	
Was/were the person(s) who is doing the experiment blinded to quality control runs?	☐	☒	☐	
**Methodology**				
Are the exposures/interventions of interest clearly described?	☒	☐	☐	
Was the sample size included in the analysis adequate?	☐	☒	☐	
Is replication of the assessment procedure possible? (description sufficiently detailed)	☒	☐	☐	
Are the distributions of principal confounders in each group of subjects to be compared clearly described?	☒	☐	☐	

Criterion	Yes	No	NA	Comments
Was there an adequate adjustment for confounding in the analyses from which the main findings were drawn?	☐	☒	☐	
**Outcomes**				
Is the hypothesis/aim/objective of the study clearly described? Must be explicit	☒	☐	☐	
Validity reported for the main outcome measure	☒	☐	☐	
**Handling Missing Data (Concurrent and Criterion Validity)**				
Compliance acceptable in all groups (80% acceptable)	☒	☐	☐	
Was the percentage of missing items given	☒	☐	☐	
Withdrawal/dropouts rate described and acceptable	☒	☐	☐	
Have the characteristics of participants lost to follow-up been described?	☒	☐	☐	
Was compliance with the intervention/s reliable?	☒	☐	☐	
Was there a description of how missing items were handled?	☒	☐	☐	
Were hypotheses regarding correlations or mean differences formulated a priori (i.e. before data collection)?	☒	☐	☐	
**Statistical Analysis**				
Have actual probability values been reported (e.g. 0.035 rather than <0.05) for the main outcomes except where the probability value is less than 0.001?	☒	☐	☐	
Sample size described for each group	☒	☐	☐	
Were design and statistical methods adequate for the hypotheses to be tested?	☐	☒	☐	
Has confidence interval for pre- and post-intervention or change in outcomes from before to after intervention been reported?	☐	☒	☐	

Criterion	Yes	No	NA	Comments
Have effect sizes for outcomes been reported or can be computed by the reviewer?	☒	☐	☐	
**Results**				
Are the main findings of the study clearly described?	☒	☐	☐	
Have all important adverse events that may be a consequence of the intervention been reported?	☒	☐	☐	

4. Title: Metagenomic Analyses Expand Bacterial and Functional Profiling Biomarkers for Colorectal Cancer in a Hainan Cohort, China

Criterion	Yes	No	NA	Comments
**Subjects recruitment**				
Are the characteristics of the participants included in the study clearly described?	☐	☒	☐	
Were the demographic characteristics of the sample reported for each group analyzed?	☐	☒	☐	
Were the subjects asked to participate in the study representative of the entire population from which they were recruited?	☐	☒	☐	
Were those subjects who were prepared to participate representative of the entire population from which they were recruited?	☐	☒	☐	
**Examiners**				
Was/were the person(s) who is doing the experiment blinded to the study groups?	☐	☒	☐	
Was/were the person(s) who is doing the experiment blinded to quality control runs?	☐	☒	☐	
**Methodology**				
Are the exposures/interventions of interest clearly described?	☐	☒	☐	
Was the sample size included in the analysis adequate?	☐	☒	☐	
Is replication of the assessment procedure possible? (description sufficiently detailed)	☒	☐	☐	

Criterion	Yes	No	NA	Comments
Are the distributions of principal confounders in each group of subjects to be compared clearly described?	☐	☒	☐	
Was there an adequate adjustment for confounding in the analyses from which the main findings were drawn?	☐	☒	☐	
**Outcomes**				
Is the hypothesis/aim/objective of the study clearly described? Must be explicit	☒	☐	☐	
Validity reported for the main outcome measure	☒	☐	☐	
**Handling Missing Data (Concurrent and Criterion Validity)**				
Compliance acceptable in all groups (80% acceptable)	☒	☐	☐	
Was the percentage of missing items given?	☒	☐	☐	
Withdrawal/dropouts rate described and acceptable	☒	☐	☐	
Have the characteristics of participants lost to follow-up been described?	☒	☐	☐	
Was compliance with the intervention/s reliable?	☒	☐	☐	
Was there a description of how missing items were handled?	☒	☐	☐	
Were hypotheses regarding correlations or mean differences formulated a priori (i.e. before data collection)?	☒	☐	☐	
**Statistical Analysis**				
Have actual probability values been reported (e.g. 0.035 rather than <0.05) for the main outcomes except where the probability value is less than 0.001?	☒	☐	☐	
Sample size described for each group?	☐	☒	☐	
Were design and statistical methods adequate for the hypotheses to be tested?	☐	☒	☐	

Criterion	Yes	No	NA	Comments
Has confidence interval for pre- and post-intervention or change in outcomes from before to after intervention been reported?	☒	☐	☐	
Have effect sizes for outcomes been reported or can be computed by the reviewer?	☒	☐	☐	
**Results**				
Are the main findings of the study clearly described?	☒	☐	☐	
Have all important adverse events that may be a consequence of the intervention been reported?	☐	☒	☐	

5. Title: Serum Trimethylamine N-oxide, Carnitine, Choline and Betaine in Relation to Colorectal Cancer Risk in the Alpha Tocopherol and Beta Carotene Study

Criterion	Yes	No	NA	Comments
**Subjects recruitment**				
Are the characteristics of the participants included in the study clearly described?	☒	☐	☐	
Were the demographic characteristics of the sample reported for each group analyzed?	☐	☒	☐	
Were the subjects asked to participate in the study representative of the entire population from which they were recruited?	☐	☒	☐	
Were those subjects who were prepared to participate representative of the entire population from which they were recruited?	☐	☒	☐	
**Examiners**				
Was/were the person(s) who is doing the experiment blinded to the study groups?	☒	☐	☐	
Was/were the person(s) who is doing the experiment blinded to quality control runs?	☒	☐	☐	
**Methodology**				
Are the exposures/interventions of interest clearly described?	☒	☐	☐	
Was the sample size included in the analysis adequate?	☒	☐	☐	
Is replication of the assessment procedure possible? (description sufficiently detailed)	☒	☐	☐	

Criterion	Yes	No	NA	Comments
Are the distributions of principal confounders in each group of subjects to be compared clearly described?	☒	☐	☐	
Was there an adequate adjustment for confounding in the analyses from which the main findings were drawn?	☒	☐	☐	
**Outcomes**				
Is the hypothesis/aim/objective of the study clearly described? Must be explicit	☒	☐	☐	
Validity reported for the main outcome measure	☒	☐	☐	
**Handling Missing Data (Concurrent and Criterion Validity)**				
Compliance acceptable in all groups (80% acceptable)	☒	☐	☐	
Was the percentage of missing items given?	☒	☐	☐	
Withdrawal/dropouts rate described and acceptable	☒	☐	☐	
Have the characteristics of participants lost to follow-up been described?	☒	☐	☐	
Was compliance with the intervention/s reliable?	☒	☐	☐	
Was there a description of how missing items were handled?	☒	☐	☐	
Were hypotheses regarding correlations or mean differences formulated a priori (i.e. before data collection)?	☒	☐	☐	
**Statistical Analysis**				
Have actual probability values been reported (e.g. 0.035 rather than <0.05) for the main outcomes except where the probability value is less than 0.001?	☒	☐	☐	
Sample size described for each group	☒	☐	☐	
Were design and statistical methods adequate for the hypotheses to be tested?	☒	☐	☐	

Criterion	Yes	No	NA	Comments
Has confidence interval for pre- and post-intervention or change in outcomes from before to after intervention been reported?	☒	☐	☐	
Have effect sizes for outcomes been reported or can be computed by the reviewer?	☒	☐	☐	
**Results**				
Are the main findings of the study clearly described?	☒	☐	☐	
Have all important adverse events that may be a consequence of the intervention been reported?	☒	☐	☐	

6 Title: Colorectal cancer diagnostic model utilizing metagenomic and metabolomic data of stool microbial extracellular vesicles

Criterion	Yes	No	NA	Comments
**Subjects recruitment**				
Are the characteristics of the participants included in the study clearly described?	☒	☐	☐	
Were the demographic characteristics of the sample reported for each group analyzed?	☒	☐	☐	
Were the subjects asked to participate in the study representative of the entire population from which they were recruited?	☒	☐	☐	
Were those subjects who were prepared to participate representative of the entire population from which they were recruited?	☒	☐	☐	
**Examiners**				
Was/were the person(s) who is doing the experiment blinded to the study groups?	☐	☒	☐	
Was/were the person(s) who is doing the experiment blinded to quality control runs?	☐	☒	☐	
**Methodology**				
Are the exposures/interventions of interest clearly described?	☐	☒	☐	
Was the sample size included in the analysis adequate?	☒	☐	☐	
Is replication of the assessment procedure possible? (description sufficiently detailed)	☒	☐	☐	
Are the distributions of principal confounders in each group of subjects to be compared clearly described?	☒	☐	☐	

Criterion	Yes	No	NA	Comments
Was there an adequate adjustment for confounding in the analyses from which the main findings were drawn?	☒	☐	☐	
**Outcomes**				
Is the hypothesis/aim/objective of the study clearly described? Must be explicit	☒	☐	☐	
Validity reported for the main outcome measure	☒	☐	☐	
**Handling Missing Data (Concurrent and Criterion Validity)**				
Compliance acceptable in all groups (80% acceptable)	☒	☐	☐	
Was the percentage of missing items given (Only for the analysis of the effect of group exercise)?	☒	☐	☐	
Withdrawal/dropouts rate described and acceptable	☒	☐	☐	
Have the characteristics of participants lost to follow-up been described?	☒	☐	☐	
Was compliance with the intervention/s reliable?	☒	☐	☐	
Was there a description of how missing items were handled?	☒	☐	☐	
Were hypotheses regarding correlations or mean differences formulated a priori (i.e. before data collection)?	☒	☐	☐	
**Statistical Analysis**				
Have actual probability values been reported (e.g. 0.035 rather than <0.05) for the main outcomes except where the probability value is less than 0.001?	☒	☐	☐	
Sample size described for each group	☒	☐	☐	
Were design and statistical methods adequate for the hypotheses to be tested?	☒	☐	☐	

Criterion	Yes	No	NA	Comments
Has confidence interval for pre- and post-intervention or change in outcomes from before to after intervention been reported?	☒	☐	☐	
Have effect sizes for outcomes been reported or can be computed by the reviewer?	☒	☐	☐	
**Results**				
Are the main findings of the study clearly described?	☒	☐	☐	
Have all important adverse events that may be a consequence of the intervention been reported?	☒	☐	☐	

7. Title: Fecal Fatty Acid Profiling as a Potential New Screening Biomarker in Patients with Colorectal Cancer

Criterion	Yes	No	NA	Comments
**Subjects recruitment**				
Are the characteristics of the participants included in the study clearly described?	☒	☐	☐	
Were the demographic characteristics of the sample reported for each group analyzed?	☒	☐	☐	
Were the subjects asked to participate in the study representative of the entire population from which they were recruited?	☒	☐	☐	
Were those subjects who were prepared to participate representative of the entire population from which they were recruited?	☒	☐	☐	
**Examiners**				
Was/were the person(s) who is doing the experiment blinded to the study groups?	☐	☒	☒	
Was/were the person(s) who is doing the experiment blinded to quality control runs?	☐	☒	☒	
**Methodology**				
Are the exposures/interventions of interest clearly described?	☒	☐	☐	
Was the sample size included in the analysis adequate?	☐	☒	☐	
Is replication of the assessment procedure possible? (description sufficiently detailed)	☒	☐	☐	
Are the distributions of principal confounders in each group of subjects to be compared clearly described?	☒	☐	☐	

Criterion	Yes	No	NA	Comments
Was there an adequate adjustment for confounding in the analyses from which the main findings were drawn?	☐	☒	☐	
**Outcomes**				
Is the hypothesis/aim/objective of the study clearly described? Must be explicit	☒	☐	☐	
Validity reported for the main outcome measure	☒	☐	☐	
**Handling Missing Data (Concurrent and Criterion Validity)**				
Compliance acceptable in all groups (80% acceptable)	☒	☐	☐	
Was the percentage of missing items given	☒	☐	☐	
Withdrawal/dropouts rate described and acceptable	☒	☐	☐	
Have the characteristics of participants lost to follow-up been described?	☒	☐	☐	
Was compliance with the intervention/s reliable?	☒	☐	☐	
Was there a description of how missing items were handled?	☒	☐	☐	
Were hypotheses regarding correlations or mean differences formulated a priori (i.e. before data collection)?	☒	☐	☐	
**Statistical Analysis**				
Have actual probability values been reported (e.g. 0.035 rather than <0.05) for the main outcomes except where the probability value is less than 0.001?	☒	☐	☐	
Sample size described for each group	☒	☐	☐	
Were design and statistical methods adequate for the hypotheses to be tested?	☐	☒	☐	
Has confidence interval for pre- and post-intervention or change in outcomes from before to after intervention been reported?	☐	☒	☐	

Criterion	Yes	No	NA	Comments
Have effect sizes for outcomes been reported or can be computed by the reviewer?	☒	☐	☐	
**Results**				
Are the main findings of the study clearly described?	☒	☐	☐	
Have all important adverse events that may be a consequence of the intervention been reported?	☐	☒	☐	

8 Title: Association of circulating short chain fatty acid levels with colorectal adenomas and colorectal cancer

Criterion	Yes	No	NA	Comments
**Subjects recruitment**				
Are the characteristics of the participants included in the study clearly described?	☒	☐	☐	
Were the demographic characteristics of the sample reported for each group analyzed?	☒	☐	☐	
Were the subjects asked to participate in the study representative of the entire population from which they were recruited?	☐	☒	☐	
Were those subjects who were prepared to participate representative of the entire population from which they were recruited?	☐	☒	☐	
**Examiners**				
Was/were the person(s) who is doing the experiment blinded to the study groups?	☐	☒	☒	
Was/were the person(s) who is doing the experiment blinded to quality control runs?	☐	☒	☒	
**Methodology**				
Are the exposures/interventions of interest clearly described?	☐	☒	☐	
Was the sample size included in the analysis adequate?	☒	☐	☐	
Is replication of the assessment procedure possible? (description sufficiently detailed)	☒	☐	☐	
Are the distributions of principal confounders in each group of subjects to be compared clearly described?	☒	☐	☐	

Criterion	Yes	No	NA	Comments
Was there an adequate adjustment for confounding in the analyses from which the main findings were drawn?	☒	☐	☐	
**Outcomes**				
Is the hypothesis/aim/objective of the study clearly described?	☒	☐	☐	
Validity reported for the main outcome measure	☒	☐	☐	
**Handling Missing Data (Concurrent and Criterion Validity)**				
Compliance acceptable in all groups (80% acceptable)	☒	☐	☐	
Was the percentage of missing items given	☒	☐	☐	
Withdrawal/dropouts rate described and acceptable	☒	☐	☐	
Have the characteristics of participants lost to follow-up been described?	☒	☐	☐	
Was compliance with the intervention/s reliable?	☐	☒	☐	
Was there a description of how missing items were handled?	☒	☐	☐	
Were hypotheses regarding correlations or mean differences formulated a priori (i.e. before data collection)?	☒	☐	☐	
**Statistical Analysis**				
Have actual probability values been reported (e.g. 0.035 rather than <0.05) for the main outcomes except where the probability value is less than 0.001?	☒	☐	☐	
Sample size described for each group	☒	☐	☐	
Were design and statistical methods adequate for the hypotheses to be tested?	☒	☐	☐	
Has confidence interval for pre- and post-intervention or change in outcomes from before to after intervention been reported?	☒	☐	☐	

Criterion	Yes	No	NA	Comments
Have effect sizes for outcomes been reported or can be computed by the reviewer?	☒	☐	☐	
**Results**				
Are the main findings of the study clearly described?	☒	☐	☐	
Have all important adverse events that may be a consequence of the intervention been reported?	☐	☒	☐	

9 Title: Assessment of oncogenic role of intestinal microbiota in colorectal cancer patients

Criterion	Yes	No	NA	Comments
**Subjects recruitment**				
Are the characteristics of the participants included in the study clearly described?	☐	☒	☐	
Were the demographic characteristics of the sample reported for each group analyzed?	☒	☐	☐	
Were the subjects asked to participate in the study representative of the entire population from which they were recruited?	☒	☐	☐	
Were those subjects who were prepared to participate representative of the entire population from which they were recruited?	☒	☐	☐	
**Examiners**				
Was/were the person(s) who is doing the experiment blinded to the study groups?	☐	☒	☐	
Was/were the person(s) who is doing the experiment blinded to quality control runs?	☐	☒	☐	
**Methodology**				
Are the exposures/interventions of interest clearly described?	☒	☐	☐	
Was the sample size included in the analysis adequate?	☒	☐	☐	
Is replication of the assessment procedure possible? (description sufficiently detailed)	☒	☐	☐	
Are the distributions of principal confounders in each group of subjects to be compared clearly described?	☐	☒	☐	

Criterion	Yes	No	NA	Comments
Was there an adequate adjustment for confounding in the analyses from which the main findings were drawn?	☐	☒	☐	
**Outcomes**				
Is the hypothesis/aim/objective of the study clearly described? Must be explicit	☒	☐	☐	
Validity reported for the main outcome measure	☒	☐	☐	
**Handling Missing Data (Concurrent and Criterion Validity)**				
Compliance acceptable in all groups (80% acceptable)	☒	☐	☐	
Was the percentage of missing items given	☒	☐	☐	
Withdrawal/dropouts rate described and acceptable	☒	☐	☐	
Have the characteristics of participants lost to follow-up been described?	☒	☐	☐	
Was compliance with the intervention/s reliable?	☐	☒	☐	
Was there a description of how missing items were handled?	☒	☐	☐	
Were hypotheses regarding correlations or mean differences formulated a priori (i.e. before data collection)?	☐	☒	☐	
**Statistical Analysis**				
Have actual probability values been reported (e.g. 0.035 rather than <0.05) for the main outcomes except where the probability value is less than 0.001?	☒	☐	☐	
Sample size described for each group	☒	☐	☐	
Were design and statistical methods adequate for the hypotheses to be tested?	☐	☒	☐	
Has confidence interval for pre- and post-intervention or change in outcomes from before to after intervention been reported?	☐	☒	☐	

Criterion	Yes	No	NA	Comments
Have effect sizes for outcomes been reported or can be computed by the reviewer?	☐	☒	☐	
**Results**				
Are the main findings of the study clearly described?	☐	☒	☐	
Have all important adverse events that may be a consequence of the intervention been reported?	☐	☒	☐	

10. Title: Altered gut metabolites and microbiota interactions are implicated in colorectal carcinogenesis and can be non-invasive diagnostic biomarkers

Criterion	Yes	No	NA	Comments
**Subjects recruitment**				
Are the characteristics of the participants included in the study clearly described?	☒	☐	☐	
Were the demographic characteristics of the sample reported for each group analyzed?	☒	☐	☐	
Were the subjects asked to participate in the study representative of the entire population from which they were recruited?	☒	☐	☐	
Were those subjects who were prepared to participate representative of the entire population from which they were recruited?	☒	☐	☐	
**Examiners**				
Was/were the person(s) who is doing the experiment blinded to the study groups?	☐	☒	☐	
Was/were the person(s) who is doing the experiment blinded to quality control runs?	☐	☒	☐	
**Methodology**				
Are the exposures/interventions of interest clearly described?	☒	☐	☐	
Was the sample size included in the analysis adequate?	☒	☐	☐	
Is replication of the assessment procedure possible? (description sufficiently detailed)	☒	☐	☐	
Are the distributions of principal confounders in each group of subjects to be compared clearly described?	☒	☐	☐	

Criterion	Yes	No	NA	Comments
Was there an adequate adjustment for confounding in the analyses from which the main findings were drawn?	☒	☐	☐	
**Outcomes**				
Is the hypothesis/aim/objective of the study clearly described? Must be explicit	☒	☐	☐	
Validity reported for the main outcome measure	☒	☐	☐	
**Handling Missing Data (Concurrent and Criterion Validity)**				
Compliance acceptable in all groups (80% acceptable)	☒	☐	☐	
Was the percentage of missing items given (Only for the analysis of the effect of group exercise)?	☒	☐	☐	
Withdrawal/dropouts rate described and acceptable	☒	☐	☐	
Have the characteristics of participants lost to follow-up been described?	☒	☐	☐	
Was compliance with the intervention/s reliable?	☒	☐	☐	
Was there a description of how missing items were handled?	☒	☐	☐	
Were hypotheses regarding correlations or mean differences formulated a priori (i.e. before data collection)?	☒	☐	☐	
Was the expected direction of correlations or mean differences included in the hypotheses?	☒	☐	☐	
**Statistical Analysis**				
Have actual probability values been reported (e.g. 0.035 rather than <0.05) for the main outcomes except where the probability value is less than 0.001?	☒	☐	☐	
Sample size described for each group	☒	☐	☐	

Criterion	Yes	No	NA	Comments
Were design and statistical methods adequate for the hypotheses to be tested?	☒	☐	☐	
Has confidence interval for pre- and post-intervention or change in outcomes from before to after intervention been reported?	☒	☐	☐	
Have effect sizes for outcomes been reported or can be computed by the reviewer?	☒	☐	☐	
**Results**				
Are the main findings of the study clearly described?	☒	☐	☐	
Have all important adverse events that may be a consequence of the intervention been reported?	☒	☐	☐	

11 Title: Fecal metabolomics: assay performance and association with colorectal cancer

Criterion	Yes	No	NA	Comments
**Subjects' recruitment**				
Are the characteristics of the participants included in the study clearly described?	☒	☐	☐	
Were the demographic characteristics of the sample reported for each group analyzed?	☒	☐	☐	
Were the subjects asked to participate in the study representative of the entire population from which they were recruited?	☐	☒	☐	
Were those subjects who were prepared to participate representative of the entire population from which they were recruited?	☐	☒	☐	
**Examiners**				
Was/were the person(s) who is doing the experiment blinded to the study groups?	☐	☒	☐	
Was/were the person(s) who is doing the experiment blinded to quality control runs?	☒	☐	☐	
**Methodology**				
Are the exposures/interventions of interest clearly described?	☐	☒	☐	
Was the sample size included in the analysis adequate?	☒	☐	☐	
Is replication of the assessment procedure possible? (description sufficiently detailed)	☒	☐	☐	
Are the distributions of principal confounders in each group of subjects to be compared clearly described?	☐	☒	☐	

Criterion	Yes	No	NA	Comments
Was there an adequate adjustment for confounding in the analyses from which the main findings were drawn?	☐	☒	☐	
**Outcomes**				
Is the hypothesis/aim/objective of the study clearly described? Must be explicit	☒	☐	☐	
Validity reported for the main outcome measure	☒	☐	☐	
**Handling Missing Data (Concurrent and Criterion Validity)**				
Compliance acceptable in all groups (80% acceptable)	☒	☐	☐	
Was the percentage of missing items given	☒	☐	☐	
Withdrawal/dropouts rate described and acceptable	☒	☐	☐	
Have the characteristics of participants lost to follow-up been described?	☒	☐	☐	
Was compliance with the intervention/s reliable?	☐	☒	☐	
Was there a description of how missing items were handled?	☒	☐	☐	
Were hypotheses regarding correlations or mean differences formulated a priori (i.e. before data collection)?	☒	☐	☐	
**Statistical Analysis**				
Have actual probability values been reported (e.g. 0.035 rather than <0.05) for the main outcomes except where the probability value is less than 0.001?	☒	☐	☐	
Sample size described for each group	☒	☐	☐	
Were design and statistical methods adequate for the hypotheses to be tested?	☐	☒	☐	
Has confidence interval for pre- and post-intervention or change in outcomes from before to after intervention been reported?	☐	☒	☐	

Criterion	Yes	No	NA	Comments
Have effect sizes for outcomes been reported or can be computed by the reviewer?	☐	☒	☐	
**Results**				
Are the main findings of the study clearly described?	☒	☐	☐	
Have all important adverse events that may be a consequence of the intervention been reported?	☐	☒	☐	

12. Title: Fecal Microbiota, Fecal Metabolome, and Colorectal Cancer Interrelations

Criterion	Yes	No	NA	Comments
**Subjects' recruitment**				
Are the characteristics of the participants included in the study clearly described?	☒	☐	☐	
Were the demographic characteristics of the sample reported for each group analyzed?	☒	☐	☐	
Were the subjects asked to participate in the study representative of the entire population from which they were recruited?	☐	☒	☐	
Were those subjects who were prepared to participate representative of the entire population from which they were recruited?	☐	☒	☐	
**Examiners**				
Was/were the person(s) who is doing the experiment blinded to the study groups?	☐	☒	☐	
Was/were the person(s) who is doing the experiment blinded to quality control runs?	☐	☒	☐	
**Methodology**				
Are the exposures/interventions of interest clearly described?	☒	☐	☐	
Was the sample size included in the analysis adequate?	☒	☐	☐	
Is replication of the assessment procedure possible? (description sufficiently detailed)	☒	☐	☐	
Are the distributions of principal confounders in each group of subjects to be compared clearly described?	☒	☐	☐	

Criterion	Yes	No	NA	Comments
Was there an adequate adjustment for confounding in the analyses from which the main findings were drawn?	☒	☐	☐	
**Outcomes**				
Is the hypothesis/aim/objective of the study clearly described? Must be explicit	☒	☐	☐	
Validity reported for the main outcome measure	☒	☐	☐	
**Handling Missing Data (Concurrent and Criterion Validity)**				
Compliance acceptable in all groups (80% acceptable)	☒	☐	☐	
Was the percentage of missing items given?	☒	☐	☐	
Withdrawal/dropouts rate described and acceptable	☒	☐	☐	
Have the characteristics of participants lost to follow-up been described?	☒	☐	☐	
Was compliance with the intervention/s reliable?	☒	☐	☐	
Was there a description of how missing items were handled?	☒	☐	☐	
Were hypotheses regarding correlations or mean differences formulated a priori (i.e. before data collection)?	☒	☐	☐	
**Statistical Analysis**				
Have actual probability values been reported (e.g. 0.035 rather than <0.05) for the main outcomes except where the probability value is less than 0.001?	☒	☐	☐	
Sample size described for each group	☒	☐	☐	
Were design and statistical methods adequate for the hypotheses to be tested?	☒	☐	☐	
Has confidence interval for pre- and post-intervention or change in outcomes from before to after intervention been reported?	☒	☐	☐	

Criterion	Yes	No	NA	Comments
Have effect sizes for outcomes been reported or can be computed by the reviewer?	☒	☐	☐	
**Results**				
Are the main findings of the study clearly described?	☒	☐	☐	
Have all important adverse events that may be a consequence of the intervention been reported?	☒	☐	☐	

13. Title: Integrative Analysis of Fecal Metagenomics and Metabolomics in Colorectal Cancer

Criterion	Yes	No	NA	Comments
**Subjects recruitment**				
Are the characteristics of the participants included in the study clearly described?	☒	☐	☐	
Were the demographic characteristics of the sample reported for each group analyzed?	☐	☒	☐	
Were the subjects asked to participate in the study representative of the entire population from which they were recruited?	☐	☒	☐	
Were those subjects who were prepared to participate representative of the entire population from which they were recruited?	☐	☒	☐	
**Examiners**				
Was/were the person(s) who is doing the experiment blinded to the study groups?	☐	☒	☐	
Was/were the person(s) who is doing the experiment blinded to quality control runs?	☐	☒	☐	
**Methodology**				
Are the exposures/interventions of interest clearly described?	☒	☐	☐	
Was the sample size included in the analysis adequate?	☒	☐	☐	
Is replication of the assessment procedure possible? (description sufficiently detailed)	☒	☐	☐	
Are the distributions of principal confounders in each group of subjects to be compared clearly described?	☒	☐	☐	

Criterion	Yes	No	NA	Comments
Was there an adequate adjustment for confounding in the analyses from which the main findings were drawn?	☒	☐	☐	
**Outcomes**				
Is the hypothesis/aim/objective of the study clearly described? Must be explicit	☒	☐	☐	
Validity reported for the main outcome measure	☒	☐	☐	
**Handling Missing Data (Concurrent and Criterion Validity)**				
Compliance acceptable in all groups (80% acceptable)	☒	☐	☐	
Was the percentage of missing items given (Only for the analysis of the effect of group exercise)?	☒	☐	☐	
Withdrawal/dropouts rate described and acceptable	☒	☐	☐	
Have the characteristics of participants lost to follow-up been described?	☒	☐	☐	
Was compliance with the intervention/s reliable?	☒	☐	☐	
Was there a description of how missing items were handled?	☒	☐	☐	
Were hypotheses regarding correlations or mean differences formulated a priori (i.e. before data collection)?	☒	☐	☐	
**Statistical Analysis**				
Have actual probability values been reported (e.g. 0.035 rather than <0.05) for the main outcomes except where the probability value is less than 0.001?	☒	☐	☐	
Sample size described for each group	☒	☐	☐	
Were design and statistical methods adequate for the hypotheses to be tested?	☒	☐	☐	

Criterion	Yes	No	NA	Comments
Has confidence interval for pre- and post-intervention or change in outcomes from before to after intervention been reported?	☒	☐	☐	
Have effect sizes for outcomes been reported or can be computed by the reviewer?	☒	☐	☐	
**Results**				
Are the main findings of the study clearly described?	☒	☐	☐	
Have all important adverse events that may be a consequence of the intervention been reported?	☒	☐	☐	

14. Title: Metabonomics Identifies Serum Metabolite Markers of Colorectal Cancer

Criterion	Yes	No	NA	Comments
**Subjects' recruitment**				
Are the characteristics of the participants included in the study clearly described?	☒	☐	☐	
Were the demographic characteristics of the sample reported for each group analyzed?	☒	☐	☐	
Were the subjects asked to participate in the study representative of the entire population from which they were recruited?	☒	☐	☐	
Were those subjects who were prepared to participate representative of the entire population from which they were recruited?	☒	☐	☐	
**Examiners**				
Was/were the person(s) who is doing the experiment blinded to the study groups?	☐	☒	☐	
Was/were the person(s) who is doing the experiment blinded to quality control runs?	☐	☒	☐	
**Methodology**				
Are the exposures/interventions of interest clearly described?	☒	☐	☐	
Was the sample size included in the analysis adequate?	☒	☐	☐	
Is replication of the assessment procedure possible? (description sufficiently detailed)	☒	☐	☐	
Are the distributions of principal confounders in each group of subjects to be compared clearly described?	☒	☐	☐	

Criterion	Yes	No	NA	Comments
Was there an adequate adjustment for confounding in the analyses from which the main findings were drawn?	☒	☐	☐	
**Outcomes**				
Is the hypothesis/aim/objective of the study clearly described? Must be explicit	☒	☐	☐	
Validity reported for the main outcome measure	☒	☐	☐	
**Handling Missing Data (Concurrent and Criterion Validity)**				
Compliance acceptable in all groups (80% acceptable)	☒	☐	☐	
Was the percentage of missing items given (Only for the analysis of the effect of group exercise)?	☒	☐	☐	
Withdrawal/dropouts rate described and acceptable	☒	☐	☐	
Have the characteristics of participants lost to follow-up been described?	☒	☐	☐	
Was compliance with the intervention/s reliable?	☒	☐	☐	
Was there a description of how missing items were handled?	☒	☐	☐	
Were hypotheses regarding correlations or mean differences formulated a priori (i.e. before data collection)?	☒	☐	☐	
**Statistical Analysis**				
Have actual probability values been reported (e.g. 0.035 rather than <0.05) for the main outcomes except where the probability value is less than 0.001?	☐	☒	☐	
Sample size described for each group	☒	☐	☐	
Were design and statistical methods adequate for the hypotheses to be tested?	☒	☐	☐	

Criterion	Yes	No	NA	Comments
Has confidence interval for pre- and post-intervention or change in outcomes from before to after intervention been reported?	☐	☒	☐	
Have effect sizes for outcomes been reported or can be computed by the reviewer?	☒	☐	☐	
**Results**				
Are the main findings of the study clearly described?	☒	☐	☐	
Have all important adverse events that may be a consequence of the intervention been reported?	☐	☐	☐	

15. Title: Tumour-associated and non-tumour-associated microbiota in colorectal cancer

Criterion	Yes	No	NA	Comments
**Subjects' recruitment**				
Are the characteristics of the participants included in the study clearly described?	☒	☐	☐	
Were the demographic characteristics of the sample reported for each group analyzed?	☒	☐	☐	
Were the subjects asked to participate in the study representative of the entire population from which they were recruited?	☒	☐	☐	
Were those subjects who were prepared to participate representative of the entire population from which they were recruited?	☒	☐	☐	
**Examiners**				
Was/were the person(s) who is doing the experiment blinded to the study groups?	☐	☒	☐	
Was/were the person(s) who is doing the experiment blinded to quality control runs?	☐	☒	☐	
**Methodology**				
Are the exposures/interventions of interest clearly described?	☒	☐	☐	
Was the sample size included in the analysis adequate?	☒	☐	☐	
Is replication of the assessment procedure possible? (description sufficiently detailed)	☒	☐	☐	
Are the distributions of principal confounders in each group of subjects to be compared clearly described?	☒	☐	☐	

Criterion	Yes	No	NA	Comments
Was there an adequate adjustment for confounding in the analyses from which the main findings were drawn?	☒	☐	☐	
**Outcomes**				
Is the hypothesis/aim/objective of the study clearly described? Must be explicit	☒	☐	☐	
Validity reported for the main outcome measure	☒	☐	☐	
**Handling Missing Data (Concurrent and Criterion Validity)**				
Compliance acceptable in all groups (80% acceptable)	☒	☐	☐	
Was the percentage of missing items given?	☒	☐	☐	
Withdrawal/dropouts rate described and acceptable	☒	☐	☐	
Have the characteristics of participants lost to follow-up been described?	☒	☐	☐	
Was compliance with the intervention/s reliable?	☒	☐	☐	
Was there a description of how missing items were handled?	☒	☐	☐	
Were hypotheses regarding correlations or mean differences formulated a priori (i.e. before data collection)?	☒	☐	☐	
Was the expected direction of correlations or mean differences included in the hypotheses?	☒	☐	☐	
**Statistical Analysis**				
Have actual probability values been reported (e.g. 0.035 rather than <0.05) for the main outcomes except where the probability value is less than 0.001?	☒	☐	☐	
Sample size described for each group	☒	☐	☐	
Were design and statistical methods adequate for the hypotheses to be tested?	☒	☐	☐	

Criterion	Yes	No	NA	Comments
Has confidence interval for pre- and post-intervention or change in outcomes from before to after intervention been reported?	☒	☐	☐	
Have effect sizes for outcomes been reported or can be computed by the reviewer?	☒	☐	☐	
**Results**				
Are the main findings of the study clearly described?	☒	☐	☐	
Have all important adverse events that may be a consequence of the intervention been reported?	☒	☐	☐	

16. Title: Potential of fecal microbiota for early-stage detection of colorectal cancer

Criterion	Yes	No	NA	Comments
**Subjects' recruitment**				
Are the characteristics of the participants included in the study clearly described?	☒	☐	☐	
Were the demographic characteristics of the sample reported for each group analyzed?	☒	☐	☐	
Were the subjects asked to participate in the study representative of the entire population from which they were recruited?	☒	☐	☐	
Were those subjects who were prepared to participate representative of the entire population from which they were recruited?	☒	☐	☐	
**Examiners**				
Was/were the person(s) who is doing the experiment blinded to the study groups?	☐	☒	☐	
Was/were the person(s) who is doing the experiment blinded to quality control runs?	☐	☒	☐	
**Methodology**				
Are the exposures/interventions of interest clearly described?	☒	☐	☐	
Was the sample size included in the analysis adequate?	☒	☐	☐	
Is replication of the assessment procedure possible? (description sufficiently detailed)	☒	☐	☐	
Are the distributions of principal confounders in each group of subjects to be compared clearly described?	☒	☐	☐	

Criterion	Yes	No	NA	Comments
Was there an adequate adjustment for confounding in the analyses from which the main findings were drawn?	☒	☐	☐	
**Outcomes**				
Is the hypothesis/aim/objective of the study clearly described? Must be explicit (Only focus on objective related to the study of the effect of group exercise).	☒	☐	☐	
Validity reported for the main outcome measure	☒	☐	☐	
**Handling Missing Data (Concurrent and Criterion Validity)**				
Compliance acceptable in all groups (80% acceptable)	☒	☐	☐	
Was the percentage of missing items given?	☒	☐	☐	
Withdrawal/dropouts rate described and acceptable	☒	☐	☐	
Have the characteristics of participants lost to follow-up been described?	☒	☐	☐	
Was compliance with the intervention/s reliable?	☒	☐	☐	
Was there a description of how missing items were handled?	☒	☐	☐	
Were hypotheses regarding correlations or mean differences formulated a priori (i.e. before data collection)?	☒	☐	☐	
**Statistical Analysis**				
Have actual probability values been reported (e.g. 0.035 rather than <0.05) for the main outcomes except where the probability value is less than 0.001?	☒	☐	☐	
Sample size described for each group	☒	☐	☐	
Were design and statistical methods adequate for the hypotheses to be tested?	☒	☐	☐	

Criterion	Yes	No	NA	Comments
Has confidence interval for pre- and post-intervention or change in outcomes from before to after intervention been reported?	☒	☐	☐	
Have effect sizes for outcomes been reported or can be computed by the reviewer?	☒	☐	☐	
**Results**				
Are the main findings of the study clearly described?	☒	☐	☐	
Have all important adverse events that may be a consequence of the intervention been reported?	☒	☐	☐	

17. Title: The Human Gut Microbiome as a Screening Tool for Colorectal Cancer

Criterion	Yes	No	NA	Comments
**Subjects' recruitment**				
Are the characteristics of the participants included in the study clearly described?	☒	☐	☐	
Were the demographic characteristics of the sample reported for each group analyzed?	☒	☐	☐	
Were the subjects asked to participate in the study representative of the entire population from which they were recruited?	☒	☐	☐	
Were those subjects who were prepared to participate representative of the entire population from which they were recruited?	☒	☐	☐	
**Examiners**				
Was/were the person(s) who is doing the experiment blinded to the study groups?	☐	☐	☒	
Was/were the person(s) who is doing the experiment blinded to quality control runs?	☐	☐	☒	
**Methodology**				
Are the exposures/interventions of interest clearly described?	☒	☐	☐	
Was the sample size included in the analysis adequate?	☒	☐	☐	
Is replication of the assessment procedure possible? (description sufficiently detailed)	☒	☐	☐	
Are the distributions of principal confounders in each group of subjects to be compared clearly described?	☐	☒	☐	

Criterion	Yes	No	NA	Comments
Was there an adequate adjustment for confounding in the analyses from which the main findings were drawn?	☐	☒	☐	
**Outcomes**				
Is the hypothesis/aim/objective of the study clearly described? Must be explicit	☒	☐	☐	
Validity reported for the main outcome measure	☒	☐	☐	
**Handling Missing Data (Concurrent and Criterion Validity)**				
Compliance acceptable in all groups (80% acceptable)	☒	☐	☐	
Was the percentage of missing items given (Only for the analysis of the effect of group exercise)?	☒	☐	☐	
Withdrawal/dropouts rate described and acceptable	☒	☐	☐	
Have the characteristics of participants lost to follow-up been described?	☒	☐	☐	
Was compliance with the intervention/s reliable?	☐	☒	☐	
Was there a description of how missing items were handled?	☒	☐	☐	
Were hypotheses regarding correlations or mean differences formulated a priori (i.e. before data collection)?	☒	☐	☐	
**Statistical Analysis**				
Have actual probability values been reported (e.g. 0.035 rather than <0.05) for the main outcomes except where the probability value is less than 0.001?	☒	☐	☐	
Sample size described for each group	☒	☐	☐	
Were design and statistical methods adequate for the hypotheses to be tested?	☒	☐	☐	

Criterion	Yes	No	NA	Comments
Has confidence interval for pre- and post-intervention or change in outcomes from before to after intervention been reported?	☒	☐	☐	
Have effect sizes for outcomes been reported or can be computed by the reviewer?	☒	☐	☐	
**Results**				
Are the main findings of the study clearly described?	☒	☐	☐	
Have all important adverse events that may be a consequence of the intervention been reported?	☒	☐	☐	

18. Title: Changes of the Intestinal Microbiota, Short Chain Fatty Acids, and Fecal pH in Patients with Colorectal Cancer

Criterion	Yes	No	NA	Comments
**Subjects recruitment**				
Are the characteristics of the participants included in the study clearly described?	☒	☐	☐	
Were the demographic characteristics of the sample reported for each group analyzed?	☒	☐	☐	
Were the subjects asked to participate in the study representative of the entire population from which they were recruited?	☒	☐	☐	
Were those subjects who were prepared to participate representative of the entire population from which they were recruited?	☒	☐	☐	
**Examiners**				
Was/were the person(s) who is doing the experiment blinded to the study groups?	☐	☒	☐	
Was/were the person(s) who is doing the experiment blinded to quality control runs?	☐	☒	☐	
**Methodology**				
Are the exposures/interventions of interest clearly described?	☒	☐	☐	
Was the sample size included in the analysis adequate?	☒	☐	☐	
Is replication of the assessment procedure possible? (description sufficiently detailed)	☒	☐	☐	
Are the distributions of principal confounders in each group of subjects to be compared clearly described?	☐	☒	☐	

Criterion	Yes	No	NA	Comments
Was there an adequate adjustment for confounding in the analyses from which the main findings were drawn?	☐	☒	☐	
**Outcomes**				
Is the hypothesis/aim/objective of the study clearly described? Must be explicit	☒	☐	☐	
Validity reported for the main outcome measure	☒	☐	☐	
**Handling Missing Data (Concurrent and Criterion Validity)**				
Compliance acceptable in all groups (80% acceptable)	☒	☐	☐	
Was the percentage of missing items given?	☒	☐	☐	
Withdrawal/dropouts rate described and acceptable	☒	☐	☐	
Have the characteristics of participants lost to follow-up been described?	☒	☐	☐	
Was compliance with the intervention/s reliable?	☐	☒	☐	
Was there a description of how missing items were handled?	☒	☐	☐	
Were hypotheses regarding correlations or mean differences formulated a priori (i.e. before data collection)?	☒	☐	☐	
**Statistical Analysis**				
Have actual probability values been reported (e.g. 0.035 rather than <0.05) for the main outcomes except where the probability value is less than 0.001?	☒	☐	☐	
Sample size described for each group	☒	☐	☐	
Were design and statistical methods adequate for the hypotheses to be tested?	☒	☐	☐	
Has confidence interval for pre- and post-intervention or change in outcomes from before to after intervention been reported?	☐	☒	☐	

Criterion	Yes	No	NA	Comments
Have effect sizes for outcomes been reported or can be computed by the reviewer?	☒	☐	☐	
**Results**				
Are the main findings of the study clearly described?	☒	☐	☐	
Have all important adverse events that may be a consequence of the intervention been reported?	☐	☒	☐	

19. Title: Bacteroides, butyric acid and t10,c12-CLA changes in colorectal adenomatous polyp patients

Criterion	Yes	No	NA	Comments
**Subjects' recruitment**				
Are the characteristics of the participants included in the study clearly described?	☒	☐	☐	
Were the demographic characteristics of the sample reported for each group analyzed?	☒	☐	☐	
Were the subjects asked to participate in the study representative of the entire population from which they were recruited?	☒	☐	☐	
Were those subjects who were prepared to participate representative of the entire population from which they were recruited?	☒	☐	☐	
**Examiners**				
Was/were the person(s) who is doing the experiment blinded to the study groups?	☐	☒	☐	
Was/were the person(s) who is doing the experiment blinded to quality control runs?	☐	☒	☐	
**Methodology**				
Are the exposures/interventions of interest clearly described?	☒	☐	☐	
Was the sample size included in the analysis adequate?	☒	☐	☐	
Is replication of the assessment procedure possible? (description sufficiently detailed)	☒	☐	☐	
Are the distributions of principal confounders in each group of subjects to be compared clearly described?	☐	☒	☐	

Criterion	Yes	No	NA	Comments
Was there an adequate adjustment for confounding in the analyses from which the main findings were drawn?	☐	☒	☐	
**Outcomes**				
Is the hypothesis/aim/objective of the study clearly described? Must be explicit	☒	☐	☐	
Validity reported for the main outcome measure	☒	☐	☐	
**Handling Missing Data (Concurrent and Criterion Validity)**				
Compliance acceptable in all groups (80% acceptable)	☒	☐	☐	
Was the percentage of missing items given	☒	☐	☐	
Withdrawal/dropouts rate described and acceptable	☒	☐	☐	
Have the characteristics of participants lost to follow-up been described?	☒	☐	☐	
Was compliance with the intervention/s reliable?	☐	☒	☐	
Was there a description of how missing items were handled?	☒	☐	☐	
Were hypotheses regarding correlations or mean differences formulated a priori (i.e. before data collection)?	☒	☐	☐	
**Statistical Analysis**				
Have actual probability values been reported (e.g. 0.035 rather than <0.05) for the main outcomes except where the probability value is less than 0.001?	☒	☐	☐	
Sample size described for each group	☒	☐	☐	
Were design and statistical methods adequate for the hypotheses to be tested?	☒	☐	☐	
Has confidence interval for change in outcomes from before to after intervention been reported?	☐	☒	☐	

Criterion	Yes	No	NA	Comments
Have effect sizes for outcomes been reported or can be computed by the reviewer?	☒	☐	☐	
**Results**				
Are the main findings of the study clearly described?	☒	☐	☐	
Have all important adverse events that may be a consequence of the intervention been reported?	☐	☒	☐	

20. Title: Cancer-associated fecal microbial markers in colorectal cancer detection

Criterion	Yes	No	NA	Comments
**Subjects recruitment**				
Are the characteristics of the participants included in the study clearly described?	☒	☐	☐	
Were the demographic characteristics of the sample reported for each group analyzed?	☒	☐	☐	
Were the subjects asked to participate in the study representative of the entire population from which they were recruited?	☒	☐	☐	
Were those subjects who were prepared to participate representative of the entire population from which they were recruited?	☒	☐	☐	
Was an attempt made to blind study subjects to the intervention they received?	☐	☒	☐	
**Examiners**				
Was/were the examiner(s) blinded to the results of the comparator test when comparing different test measurements?	☐	☒	☒	
Was/were the rater(s) blinded to the results of previous measurements performed by the same or different examiner(s) (e.g. blinded to pre-enrollment condition)?	☐	☒	☒	
**Methodology**				
Are the exposures/interventions of interest clearly described?	☒	☐	☐	
Was the sample size included in the analysis adequate?	☒	☐	☐	
Is replication of the assessment procedure possible? (description sufficiently detailed)	☒	☐	☐	

Criterion	Yes	No	NA	Comments
Are the distributions of principal confounders in each group of subjects to be compared clearly described?	☒	☐	☐	
Was there an adequate adjustment for confounding in the analyses from which the main findings were drawn?	☒	☐	☐	
**Outcomes**				
Is the hypothesis/aim/objective of the study clearly described? Must be explicit	☒	☐	☐	
Validity reported for the main outcome measure	☒	☐	☐	
**Handling Missing Data (Concurrent and Criterion Validity)**				
Compliance acceptable in all groups (80% acceptable)	☒	☐	☐	
Was the percentage of missing items given	☒	☐	☐	
Withdrawal/dropouts rate described and acceptable	☒	☐	☐	
Have the characteristics of participants lost to follow-up been described?	☒	☐	☐	
Was compliance with the intervention/s reliable?	☒	☐	☐	
Was there a description of how missing items were handled?	☒	☐	☐	
Were hypotheses regarding correlations or mean differences formulated a priori (i.e. before data collection)?	☒	☐	☐	
**Statistical Analysis**				
Have actual probability values been reported (e.g. 0.035 rather than <0.05) for the main outcomes except where the probability value is less than 0.001?	☒	☐	☐	
Sample size described for each group	☒	☐	☐	
Were design and statistical methods adequate for the hypotheses to be tested?	☒	☐	☐	

Criterion	Yes	No	NA	Comments
Has confidence interval for change in outcomes from before to after intervention been reported?	☐	☒	☐	
Have effect sizes for outcomes been reported or can be computed by the reviewer?	☒	☐	☐	
**Results**				
Are the main findings of the study clearly described?	☒	☐	☐	
Have all important adverse events that may be a consequence of the intervention been reported?	☒	☐	☐	

21. Title: Gut microbiota dysbiosis signature is associated with the colorectal carcinogenesis sequence and improves the diagnosis of colorectal lesions

Criterion	Yes	No	NA	Comments
**Subjects recruitment**				
Are the characteristics of the participants included in the study clearly described?	☐	☒	☐	
Were the demographic characteristics of the sample reported for each group analyzed?	☐	☒	☐	
Were the subjects asked to participate in the study representative of the entire population from which they were recruited?	☒	☐	☐	
Were those subjects who were prepared to participate representative of the entire population from which they were recruited?	☒	☐	☐	
Was an attempt made to blind study subjects to the intervention they received?	☐	☒	☐	
**Examiners**				
Was/were the examiner(s) blinded to the results of the comparator test when comparing different test measurements?	☐	☒	☒	
Was/were the rater(s) blinded to the results of previous measurements performed by the same or different examiner(s) (e.g. blinded to pre-enrollment condition)?	☐	☒	☒	
**Methodology**				
Are the exposures/interventions of interest clearly described?	☒	☒	☐	
Was the sample size included in the analysis adequate?	☒	☒	☐	
Is replication of the assessment procedure possible? (description sufficiently detailed)	☐	☒	☐	

Criterion	Yes	No	NA	Comments
Are the distributions of principal confounders in each group of subjects to be compared clearly described?	☐	☒	☐	
Was there an adequate adjustment for confounding in the analyses from which the main findings were drawn?	☐	☒	☐	
**Outcomes**				
Is the hypothesis/aim/objective of the study clearly described? Must be explicit	☒	☐	☐	
Validity reported for the main outcome measure	☒	☐	☐	
**Handling Missing Data (Concurrent and Criterion Validity)**				
Compliance acceptable in all groups (80% acceptable)	☒	☐	☐	
Was the percentage of missing items given	☒	☐	☐	
Withdrawal/dropouts rate described and acceptable	☒	☐	☐	
Have the characteristics of participants lost to follow-up been described?	☒	☐	☐	
Was compliance with the intervention/s reliable?	☒	☐	☐	
Was there a description of how missing items were handled?	☒	☐	☐	
Were hypotheses regarding correlations or mean differences formulated a priori (i.e. before data collection)?	☒	☐	☐	
**Statistical Analysis**				
Have actual probability values been reported (e.g. 0.035 rather than <0.05) for the main outcomes except where the probability value is less than 0.001?	☒	☐	☐	
Sample size described for each group	☒	☐	☐	
Were design and statistical methods adequate for the hypotheses to be tested?	☐	☒	☐	

Criterion	Yes	No	NA	Comments
Has confidence interval for change in outcomes from before to after intervention been reported?	☐	☒	☐	
Have effect sizes for outcomes been reported or can be computed by the reviewer?	☐	☒	☐	
**Results**				
Are the main findings of the study clearly described?	☒	☐	☐	
Have all important adverse events that may be a consequence of the intervention been reported?	☐	☒	☐	

22. Title: The Level Short Chain Fatty Acids and HSP 70 in Colorectal Cancer and Non-Colorectal Cancer

Criterion	Yes	No	NA	Comments
**Subjects recruitment**				
Are the characteristics of the participants included in the study clearly described?	☐	☒	☐	
Were the demographic characteristics of the sample reported for each group analyzed?	☐	☒	☐	
Were the subjects asked to participate in the study representative of the entire population from which they were recruited?	☒	☐	☐	
Were those subjects who were prepared to participate representative of the entire population from which they were recruited?	☒	☐	☐	
Was an attempt made to blind study subjects to the intervention they received?	☐	☒	☐	
**Examiners**				
Was/were the examiner(s) blinded to the results of the comparator test when comparing different test measurements?	☐	☒	☒	
Was/were the rater(s) blinded to the results of previous measurements performed by the same or different examiner(s) (e.g. blinded to pre-enrollment condition)?	☐	☒	☒	
**Methodology**				
Are the exposures/interventions of interest clearly described?	☐	☒	☐	
Was the sample size included in the analysis adequate?	☐	☒	☐	
Is replication of the assessment procedure possible? (description sufficiently detailed)	☐	☒	☐	

Criterion	Yes	No	NA	Comments
Are the distributions of principal confounders in each group of subjects to be compared clearly described?	☐	☒	☐	
Was there an adequate adjustment for confounding in the analyses from which the main findings were drawn?	☐	☒	☐	
**Outcomes**				
Is the hypothesis/aim/objective of the study clearly described? Must be explicit	☒	☐	☐	
Validity reported for the main outcome measure	☒	☐	☐	
**Handling Missing Data (Concurrent and Criterion Validity)**				
Compliance acceptable in all groups (80% acceptable)	☒	☐	☐	
Was the percentage of missing items given	☒	☐	☐	
Withdrawal/dropouts rate described and acceptable	☒	☐	☐	
Have the characteristics of participants lost to follow-up been described?	☒	☐	☐	
Was compliance with the intervention/s reliable?	☒	☐	☐	
Was there a description of how missing items were handled?	☒	☐	☐	
Were hypotheses regarding correlations or mean differences formulated a priori (i.e. before data collection)?	☒	☐	☐	
**Statistical Analysis**				
Have actual probability values been reported (e.g. 0.035 rather than <0.05) for the main outcomes except where the probability value is less than 0.001?	☒	☐	☐	
Sample size described for each group	☒	☐	☐	
Were design and statistical methods adequate for the hypotheses to be tested?	☐	☒	☐	

Criterion	Yes	No	NA	Comments
Has confidence interval for change in outcomes from before to after intervention been reported?	☐	☒	☐	
Have effect sizes for outcomes been reported or can be computed by the reviewer?	☐	☒	☐	
**Results**				
Are the main findings of the study clearly described?	☒	☐	☐	
Have all important adverse events that may be a consequence of the intervention been reported?	☐	☒	☐	

23. Title: Stool Microbiome and Metabolome Differences between Colorectal Cancer Patients and Healthy Adults

Criterion	Yes	No	NA	Comments
**Subjects recruitment**				
Are the characteristics of the participants included in the study clearly described?	☒	☐	☐	
Were the demographic characteristics of the sample reported for each group analyzed?	☒	☐	☐	
Were the subjects asked to participate in the study representative of the entire population from which they were recruited?	☒	☐	☐	
Were those subjects who were prepared to participate representative of the entire population from which they were recruited?	☒	☐	☐	
Was an attempt made to blind study subjects to the intervention they received?	☒	☐	☐	
**Examiners**				
Was/were the examiner(s) blinded to the results of the comparator test when comparing different test measurements?	☐	☒	☒	
Was/were the rater(s) blinded to the results of previous measurements performed by the same or different examiner(s) (e.g. blinded to pre-enrollment condition)?	☐	☒	☒	
**Methodology**				
Are the exposures/interventions of interest clearly described?	☒	☐	☐	
Was the sample size included in the analysis adequate?	☐	☒	☐	
Is replication of the assessment procedure possible? (description sufficiently detailed)	☒	☐	☐	

Criterion	Yes	No	NA	Comments
Are the distributions of principal confounders in each group of subjects to be compared clearly described?	☐	☒	☐	
Was there an adequate adjustment for confounding in the analyses from which the main findings were drawn?	☐	☒	☐	
**Outcomes**				
Is the hypothesis/aim/objective of the study clearly described? Must be explicit	☒	☐	☐	
Validity reported for the main outcome measure	☒	☐	☐	
**Handling Missing Data (Concurrent and Criterion Validity)**				
Compliance acceptable in all groups (80% acceptable)	☒	☐	☐	
Was the percentage of missing items given	☒	☐	☐	
Withdrawal/dropouts rate described and acceptable	☒	☐	☐	
Have the characteristics of participants lost to follow-up been described?	☒	☐	☐	
Was compliance with the intervention/s reliable?	☒	☐	☐	
Was there a description of how missing items were handled?	☒	☐	☐	
Were hypotheses regarding correlations or mean differences formulated a priori (i.e. before data collection)?	☒	☐	☐	
**Statistical Analysis**				
Have actual probability values been reported (e.g. 0.035 rather than <0.05) for the main outcomes except where the probability value is less than 0.001?	☒	☐	☐	
Sample size described for each group	☒	☐	☐	
Were design and statistical methods adequate for the hypotheses to be tested?	☐	☒	☐	

Criterion	Yes	No	NA	Comments
Has confidence interval for change in outcomes from before to after intervention been reported?	☐	☒	☐	
Have effect sizes for outcomes been reported or can be computed by the reviewer?	☐	☒	☐	
**Results**				
Are the main findings of the study clearly described?	☒	☐	☐	
Have all important adverse events that may be a consequence of the intervention been reported?	☒	☐	☐	

24. Title: Integrated microbiome and metabolome analysis reveals a novel interplay between commensal bacteria and metabolites in colorectal cancer

Criterion	Yes	No	NA	Comments
**Subjects recruitment**				
Are the characteristics of the participants included in the study clearly described?	☒	☐	☐	
Were the demographic characteristics of the sample reported for each group analyzed?	☒	☐	☐	
Were the subjects asked to participate in the study representative of the entire population from which they were recruited?	☒	☐	☐	
Were those subjects who were prepared to participate representative of the entire population from which they were recruited?	☒	☐	☐	
Was an attempt made to blind study subjects to the intervention they received?	☒	☐	☐	
**Examiners**				
Was/were the examiner(s) blinded to the results of the comparator test when comparing different test measurements?	☐	☒	☒	
Was/were the rater(s) blinded to the results of previous measurements performed by the same or different examiner(s) (e.g. blinded to pre-enrollment condition)?	☐	☒	☒	
**Methodology**				
Are the exposures/interventions of interest clearly described?	☒	☐	☐	
Was the sample size included in the analysis adequate?	☒	☐	☐	
Is replication of the assessment procedure possible? (description sufficiently detailed)	☒	☐	☐	

Criterion	Yes	No	NA	Comments
Are the distributions of principal confounders in each group of subjects to be compared clearly described?	☒	☐	☐	
Was there an adequate adjustment for confounding in the analyses from which the main findings were drawn?	☒	☐	☐	
**Outcomes**				
Is the hypothesis/aim/objective of the study clearly described? Must be explicit	☒	☐	☐	
Validity reported for the main outcome measure	☒	☐	☐	
**Handling Missing Data (Concurrent and Criterion Validity)**				
Compliance acceptable in all groups (80% acceptable)	☒	☐	☐	
Was the percentage of missing items given	☒	☐	☐	
Withdrawal/dropouts rate described and acceptable	☒	☐	☐	
Have the characteristics of participants lost to follow-up been described?	☒	☐	☐	
Was compliance with the intervention/s reliable?	☒	☐	☐	
Was there a description of how missing items were handled?	☒	☐	☐	
Were hypotheses regarding correlations or mean differences formulated a priori (i.e. before data collection)?	☒	☐	☐	
**Statistical Analysis**				
Have actual probability values been reported (e.g. 0.035 rather than <0.05) for the main outcomes except where the probability value is less than 0.001?	☒	☐	☐	
Sample size described for each group	☒	☐	☐	
Were design and statistical methods adequate for the hypotheses to be tested?	☒	☐	☐	

Criterion	Yes	No	NA	Comments
Has confidence interval for change in outcomes from before to after intervention been reported?	☐	☒	☐	
Have effect sizes for outcomes been reported or can be computed by the reviewer?	☒	☐	☐	
**Results**				
Are the main findings of the study clearly described?	☒	☐	☐	
Have all important adverse events that may be a consequence of the intervention been reported?	☒	☐	☐	
